# CSF1R signaling is a regulator of pathogenesis in progressive MS

**DOI:** 10.1038/s41419-020-03084-7

**Published:** 2020-10-23

**Authors:** Nellwyn Hagan, John L. Kane, Deepak Grover, Lisa Woodworth, Charlotte Madore, Jacqueline Saleh, Jose Sancho, Jinyu Liu, Yi Li, Jonathan Proto, Matija Zelic, Amy Mahan, Michael Kothe, Andrew A. Scholte, Maria Fitzgerald, Barbara Gisevius, Aiden Haghikia, Oleg Butovsky, Dimitry Ofengeim

**Affiliations:** 1 Sanofi, Neuroscience, 49 New York Ave, Framingham, MA 01701 USA; 2 Sanofi, Integrated Drug Discovery, 153 2nd Ave, Waltham, MA 02451 USA; 3 Sanofi, Translational Sciences, 49 New York Ave, Framingham, MA 01701 USA; 4 Ann Romney Center for Neurologic Diseases, Department of Neurology, Brigham and Women’s Hospital, Harvard Medical School, Boston, MA USA; 5 Sanofi, Drug Metabolism and Pharmacokinetics, 153 2nd Ave, Waltham, MA 02451 USA; 6 Department of Neurology, Ruhr University Bochum, St. Josef Hospital Bochum, Bochum, 44791 Germany

**Keywords:** Physiology, Multiple sclerosis

## Abstract

Microglia serve as the innate immune cells of the central nervous system (CNS) by providing continuous surveillance of the CNS microenvironment and initiating defense mechanisms to protect CNS tissue. Upon injury, microglia transition into an activated state altering their transcriptional profile, transforming their morphology, and producing pro-inflammatory cytokines. These activated microglia initially serve a beneficial role, but their continued activation drives neuroinflammation and neurodegeneration. Multiple sclerosis (MS) is a chronic, inflammatory, demyelinating disease of the CNS, and activated microglia and macrophages play a significant role in mediating disease pathophysiology and progression. Colony-stimulating factor-1 receptor (CSF1R) and its ligand CSF1 are elevated in CNS tissue derived from MS patients. We performed a large-scale RNA-sequencing experiment and identified CSF1R as a key node of disease progression in a mouse model of progressive MS. We hypothesized that modulating microglia and infiltrating macrophages through the inhibition of CSF1R will attenuate deleterious CNS inflammation and reduce subsequent demyelination and neurodegeneration. To test this hypothesis, we generated a novel potent and selective small-molecule CSF1R inhibitor (sCSF1R_inh_) for preclinical testing. sCSF1R_inh_ blocked receptor phosphorylation and downstream signaling in both microglia and macrophages and altered cellular functions including proliferation, survival, and cytokine production. In vivo, CSF1R inhibition with sCSF1R_inh_ attenuated neuroinflammation and reduced microglial proliferation in a murine acute LPS model. Furthermore, the sCSF1R_inh_ attenuated a disease-associated microglial phenotype and blocked both axonal damage and neurological impairments in an experimental autoimmune encephalomyelitis (EAE) model of MS. While previous studies have focused on microglial depletion following CSF1R inhibition, our data clearly show that signaling downstream of this receptor can be beneficially modulated in the context of CNS injury. Together, these data suggest that CSF1R inhibition can reduce deleterious microglial proliferation and modulate microglial phenotypes during neuroinflammatory pathogenesis, particularly in progressive MS.

## Introduction

Multiple sclerosis (MS) is a complex, inflammatory, demyelinating disease of the central nervous system (CNS) driven by both environmental and genetic factors^1,2^. Although no disease-causing mutations have been identified, the genes associated with this disorder point to a strong immune component which mediates disease pathophysiology^3^. As such, all currently available MS therapies target the peripheral immune system. Interestingly, the neuro-immune axis within the CNS is also disrupted in disease and presents a unique opportunity to directly modulate neuroinflammation for clinical benefit^4^. In particular, activated microglia and macrophages play a significant role in mediating disease pathophysiology and progression. Histological studies have demonstrated that microglia and macrophages accumulate in regions of active demyelination and neurodegeneration in MS^5,6^. These cells downregulate genes associated with homeostatic microglial function and adopt a more pro-inflammatory phenotype, expressing genes linked to phagocytosis, antigen presentation, and oxidative injury^7^. This aberrant microglial function in the context of disease may not only lead to demyelination but also synaptic loss and cognitive dysfunction^8^. Even microglia in the normal-appearing white matter (NAWM) of the MS brain exhibit an altered phenotype, losing homeostatic markers like P2RY12 and upregulating disease-associated markers such as CD68 and p22phox^7^. This shift in microglial phenotype becomes more pronounced with age and disease duration^7^.

Recent advances in neuroimaging technology have allowed the visualization of neuroinflammation within the brain of living MS patients and have identified a persistent phase rim around demyelinated MS lesions. This phase rim corresponds to iron-laden inflammatory microglia and macrophages around the lesion edge that continue to smolder as disease progresses^9,10^. Translocator protein (TSPO) PET ligands have been developed as a biomarker for neuroinflammation. TSPO PET imaging also shows widespread neuroinflammation in the NAWM as well as in active lesions in MS. Interestingly, this increase in TSPO PET signal is positively correlated with markers of disease progression such as EDSS score and timed 25-foot walk and negatively correlated with brain parenchymal volume^11–13^.

Colony-stimulating factor 1 receptor (CSF1R) is a key signaling node in microglia and macrophage biology. In fact, CSF1R signaling is not only required for the proper development of these cell populations but is also a critical regulator of their homeostasis in adulthood^14^. In particular, CSF1R signaling modulates proliferation, migration, differentiation, and survival of microglia and macrophages in health and disease^15–18^. Single-cell transcriptomic analyses of several preclinical murine models of neuroinflammation demonstrate that CSF1, a ligand for CSF1R, is upregulated in disease-associated microglia^19,20^. Moreover, a novel CSF1R PET ligand revealed elevated CSF1R expression in murine models of neuroinflammation as well as post-mortem brain tissue from patients with Alzheimer’s disease^21^. We hypothesize that modulating microglia and infiltrating macrophages through CSF1R inhibition will attenuate deleterious CNS inflammation and inhibit subsequent demyelination and neurodegeneration. Although the functional consequence of inhibiting CSF1R in patients is not fully understood, several preclinical models of neurodegeneration support the notion that inhibiting this pathway is beneficial in altering neuroinflammation^22–32^.

In this study, we demonstrated an upregulation of CSF1R signaling components in CNS tissue derived from progressive MS patients. Furthermore, we generated a CNS-penetrant, selective and potent CSF1R inhibitor (sCSF1R_inh_) for preclinical testing. Through a series of in vitro assays, we demonstrated that sCSF1R_inh_ abrogates CSF1R phosphorylation and downstream signaling and attenuates the inflammatory response in microglia and macrophages. In vivo, sCSF1R_inh_ reduced the number of microglia and infiltrating macrophages in an acute LPS model. In this in vivo model, CSF1R inhibition diminished microglial/macrophage proliferation and led to an attenuated inflammatory response. sCSF1R_inh_ also suppressed a deleterious microglial response in the C57BL/6 EAE (experimental autoimmune encephalomy) model as well as the MOG peptide-induced non-obese diabetic EAE model of progressive MS (NOD-EAE) and improved neurological impairments in both models. Our data point to a critical role for CSF1-mediated signaling in disease progression in MS, and show that inhibition of CSF1R signaling can be beneficial in various models of MS. Together, these data suggest that CSF1R represents a therapeutic node by which we can target detrimental neuroinflammation to reset the CNS milieu in progressive MS.

## Results

### CSF1R signaling is upregulated in progressive MS

**Fig. 1:**
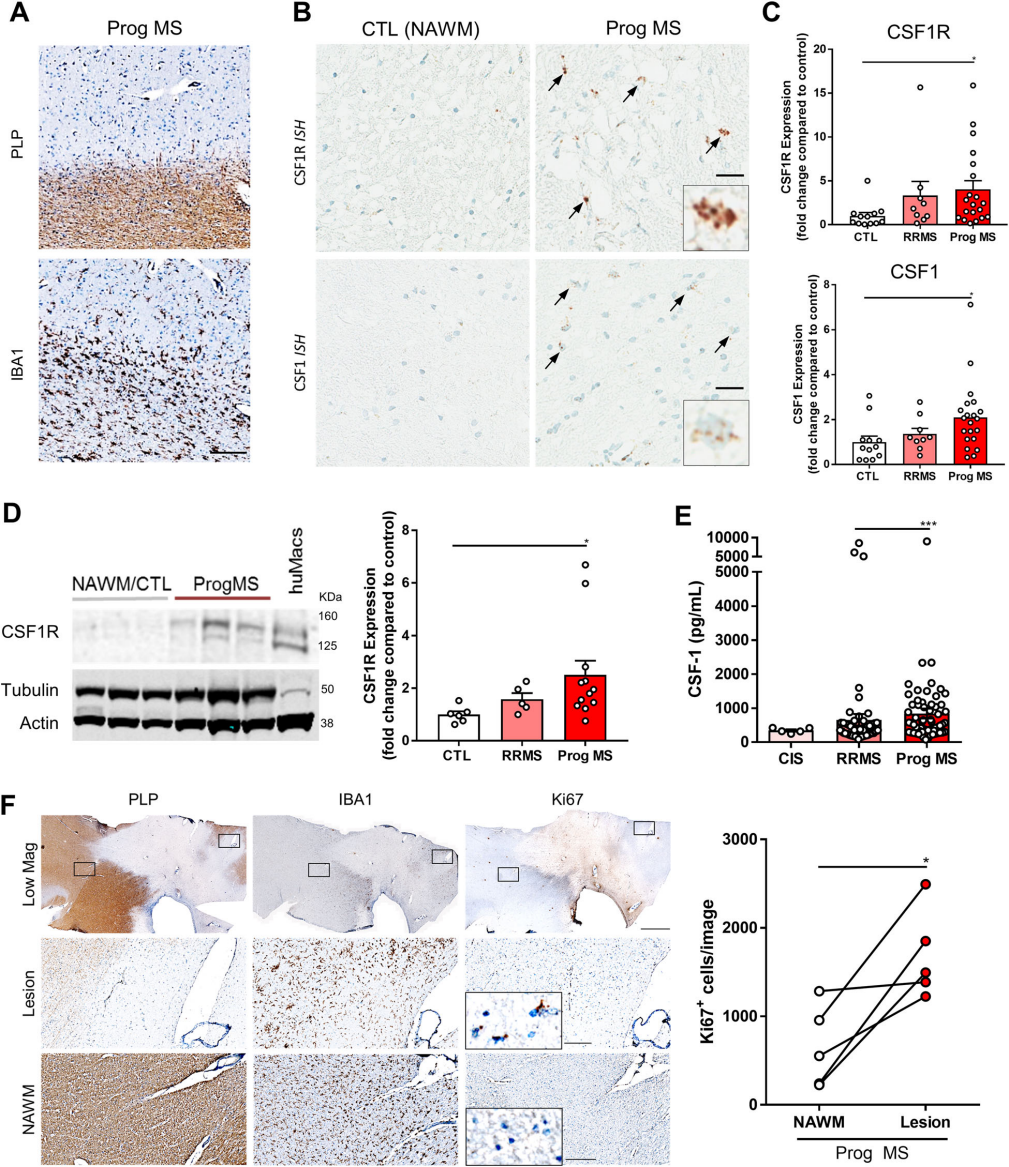
CSF1R signaling is upregulated in progressive MS. **a** Immunohistochemistry with an antibody against PLP demarks regions of demyelination (PLP^-^lesions) within the MS patient brain. Iba1 immunohistochemistry labels resident microglia and infiltrating macrophages in and around these PLP^-^ lesions. Scale bar: 200 µm **b** In situ hybridization with a CSF1R and CSF1 probe reveals the presence of these transcripts around the MS lesion. Qualitative assessment with in situ hybridization suggests that CSF1R and CSF1 transcripts are elevated in the MS patient brain as compared to the NAWM of non-MS controls. Scale bar: 50 µm. **c** Transcriptional analysis via qPCR reveals a significant increase in both CSF1R and CSF1 mRNA in the Prog MS patient brain as compared with RRMS and CTL samples. Each data point represents an individual human sample while graphical columns represent the mean and standard error. Statistical significance was determined by a one-way ANOVA and *p* values are indicated by *p < 0.05. **d** Protein expression was assessed by Western Blot with an antibody against CSF1R. CSF1R protein was elevated in Prog MS patients. Each data point represents an individual human sample while graphical columns represent the mean and standard error. Statistical significance was determined by a one-way ANOVA and *p* values are indicated by *p < 0.05. **e** CSF1 levels are also elevated in cerebral spinal fluid from Prog MS patients as compared with RRMS and CTL samples. Each data point represents an individual human sample while graphical columns represent the mean and standard error. Statistical significance was determined by a one-way ANOVA and *p* values are indicated by ***p < 0.001. **f** Immunohistochemistry with antibodies against PLP and IBA1 identify regions of demyelination and microgliosis respectively within the progressive MS patient brain and Ki67 demarks cells undergoing proliferation in the region. Scale bar: 2000 µm (top row), 200 µm (bottom two rows).

CSF1R is a type III receptor tyrosine kinase expressed on microglia and macrophages. Elevated signaling downstream of CSF1R has been implicated in deleterious microglial function in various neurodegenerative diseases^33–35^. To understand CSF1R signaling in MS, we examined CSF1R and CSF1 expression in post-mortem brain tissue from progressive MS patients as well as NAWM from non-MS control subjects. Using immunohistochemistry, we identified demyelinated (PLP^-^) cortical white matter lesions within the progressive MS patient brain (Fig. 1a). With in situ hybridization, we observed a qualitative increase in both CSF1R and CSF1 transcript levels in and around these demyelinating lesions (Fig. 1a, b). CSF1R expression was restricted to IBA1^+^ microglia and infiltrating macrophages (Supplementary Fig. 1B). To further understand the change in CSF1R signaling, we performed quantitative PCR analysis on brain tissue from relapsing-remitting MS (RRMS) and progressive MS patients as well as NAWM from non-MS controls. Although CSF1R and CSF1 mRNA levels were only modestly increased in RRMS patient samples, CSF1R and CSF1 transcripts were significantly elevated in progressive forms of the disease as compared to NAWM from control subjects (Fig. 1c). To confirm that these transcriptional changes correlated with protein levels, we also performed western blot analysis on these samples. CSF1R protein was significantly increased in progressive disease as compared to NAWM control tissue (Fig. 1d). Furthermore, we identified elevated CSF1 protein in cerebrospinal fluid derived from progressive MS patients (Fig. 1e). Interestingly, another CSF1R ligand, IL-34, was not increased in either MS tissue or cerebrospinal fluid (Supplementary Fig. 1C).

Our data clearly indicate that the CSF1R signaling pathway is active in progressive MS. Previous studies have demonstrated that CSF1 increases the rate of protein synthesis and cellular proliferation in a dose-dependent manner^36^. CSF1 achieves this effect by binding to CSF1R and activating multiple divergent downstream signaling pathways^18^. To examine whether there was increased proliferation in MS lesions, we used Ki67 expression as a surrogate of cellular proliferation in *post-mortem* brain tissue. By immunohistochemistry, we found an increase in Ki67 staining within the lesion (PLP^-^) colocalized with Iba1^+^ cells in the progressive MS brain (Fig. 1f). These results are consistent with published studies describing an increase in microglia/macrophage proliferation in MS tissue^37–39^ and suggest that CSF1R signaling may be contributing to this phenomenon. Together these data reveal an upregulation in CSF1R signaling and microglial/macrophage proliferation that may be targeted therapeutically to ameliorate neuroinflammation in progressive MS.

### CSF1R signaling is upregulated in preclinical models of MS

**Fig. 2:**
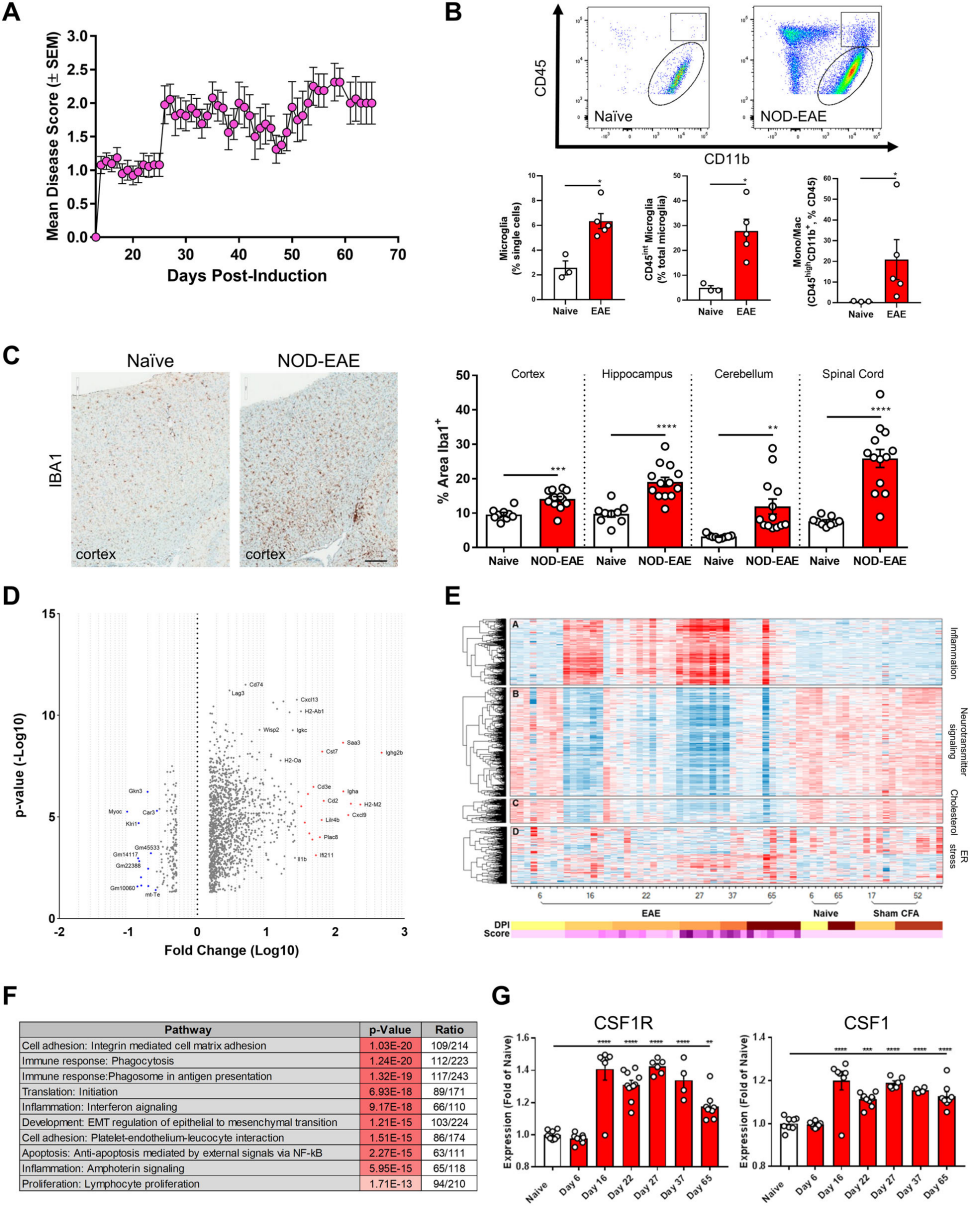
CSF1R is a key signaling node in the NOD-EAE model of progressive MS. **a** Clinical disease scores quantify the paralytic phenotype and reveal the progressive disease course of the MOG_35-55_-induced NOD-EAE model of prog MS. Data points represent the mean clinical disease score on each day and error bars represent the standard error of the mean (*n* = 26). **b** Microglia and infiltrating macrophages from the spinal cord of naïve and NOD-EAE mice were analyzed via flow cytometry. Based upon CD45 and CD11b immunoreactivity, the number of microglia (CD45^low/int^, CD11b^+^), reactive microglia (CD45^int^, CD11b^+^) and infiltrating macrophages (CD45^high^, CD11b^+^) is significantly elevated in the model. Each data point represents quantification from a single animal while graphical columns represent the mean and standard error (*n* = 3–4 per group). Statistical significance was determined by a Mann–Whitney U Test and *p* values are indicated by *p < 0.05. **c** Immunohistochemistry with an antibody against Iba1 also shows an increase in microglia and infiltrating macrophages within the cortex of NOD-EAE model as compared to naïve controls. Automated quantification of percent Iba1^+^ area with Definiens Tissue Studio Software demonstrated a significant increase in microglia/macrophage coverage in four different regions of the CNS (cortex, hippocampus, cerebellum, and spinal cord). Each data point represents the Iba1^+^ area quantified from two sections/animal while graphical columns represent the mean and standard error (*n* = 9 for naïve, *n* = 13 for NOD-EAE). Statistical significance was determined by a one-way ANOVA and *p* values are indicated by **p ≤ 0.01, *** *p* ≤ 0.001, and ****p ≤ 0.0001. Scale bar: 200 µm. **d** Volcano plot of RNA-sequencing data shows the most significantly upregulated and downregulated genes in naïve versus peak NOD-EAE spinal cord. **e** RNA-sequencing of spinal cord from different stages of the NOD-EAE model identified four key signaling modules: inflammation, neurotransmitter signaling, cholesterol, and ER stress. **f** Pathway analysis of RNA-sequencing data shows multiple immune pathways that are significantly modulated in the NOD-EAE model. **f** RNA-sequencing reveals the increase in CSF1R and CSF1 over the course of the NOD-EAE model. Data points represent the expression level for each animal and graphical columns represent the mean and standard error of these biological replicates. Statistical significance was determined by a one-way ANOVA and *p* values are indicated by **p < 0.01, ***p < 0.001, and ****p < 0.0001.

In order to investigate the role of CSF1R in progressive MS, we utilized the NOD-EAE mouse model^40,41^. This model has several unique features including a chronic-progressive clinical profile, pathological similarity to MS lesions, early axonal injury and evidence of disease activity along the whole neuro-axis that enables the examination of activated microglia and macrophages in both the spinal cord and the cerebellum^40^. We employed the NOD-EAE model to better understand the role of neuroinflammation at different stages of disease progression. We induced EAE in a large cohort of animals and quantified their clinical disease course. Similar to previous studies there was an initial peak in disease around 16 days post-induction (dpi) followed by a slight remission around 21 dpi and a subsequent chronic-progressive phase (Fig. 2a). We quantified the number of microglia and infiltrating macrophages by either flow cytometry at 42 dpi or Iba1 immunohistochemistry at 62 dpi. In naïve animals, microglia (CD45^low^CD11b^+^) represented the majority of CD45^+^ cells in the spinal cord (Fig. 2b, Supplementary Fig. 2A). Following disease induction, the number of microglia (CD45^int^CD11b^+^) as well as infiltrating macrophages (CD45^high^CD11b^+^) dramatically increased (Fig. 2b, Supplementary Fig. 2A). This enhancement in microglia/macrophages was also observed in the brain (cortex, hippocampus, cerebellum) and spinal cord by immunohistochemical analysis (Fig. 2c).

To better understand the mechanism of progression in this model, we performed RNA-sequencing on spinal cord tissue at different time points following NOD-EAE induction (Fig. 2d–f, Supplementary Fig. 2B). After performing co-expression analysis, we identified four key signaling modules: one inflammation module with a clear pattern of increased expression during disease, two modules with downregulated expression corresponding to neuronal function and cholesterol biosynthesis, and a final module with a varied pattern of expression identified as an ER stress response module (Fig. 2e). Interestingly, within the cholesterol pathway, we identified several alterations in the sterol synthesis pathway that are differentially regulated during NOD-EAE (data not shown) and linked to myelination in MS^42^. Although changes in the neuronal and cholesterol biosynthesis modules correlate with disease status in the NOD-EAE model, the strongest transcriptional signature was mediated by the inflammatory response and a key regulator of these changes was identified as CSF1R. In particular, CSF1R and CSF1 mRNA expression increased with disease progression, mirroring the pattern of clinical disease scores in the model (Fig. 2g).

Our RNA-sequencing data in the NOD-EAE model identified a strong CNS immune component and highlighted the CSF1R signaling pathway in disease progression. These data are consistent with animal studies showing a marked increase in CSF1 mRNA levels in microglia from Amyotrophic Lateral Sclerosis (ALS), Alzheimer’s Disease (AD), and MS preclinical mouse models (Supplementary Fig. 1D). Both in vitro and in vivo studies suggest that CSF1 upregulation is, at least in part, due to a microglial inflammatory response (Supplementary Fig. 1D) and that CSF1-dependent CSF1R activation may be driving deleterious neuroinflammation in disease. Similar to the increased expression levels of the CSF1R signaling complex, these results suggest that there is increased activation of this receptor in disease progression. Thus, therapeutically targeting CSF1R may reduce detrimental neuroinflammation and halt disease progression in MS.

### Generation of a potent and selective CSF1R inhibitor

**Fig. 3:**
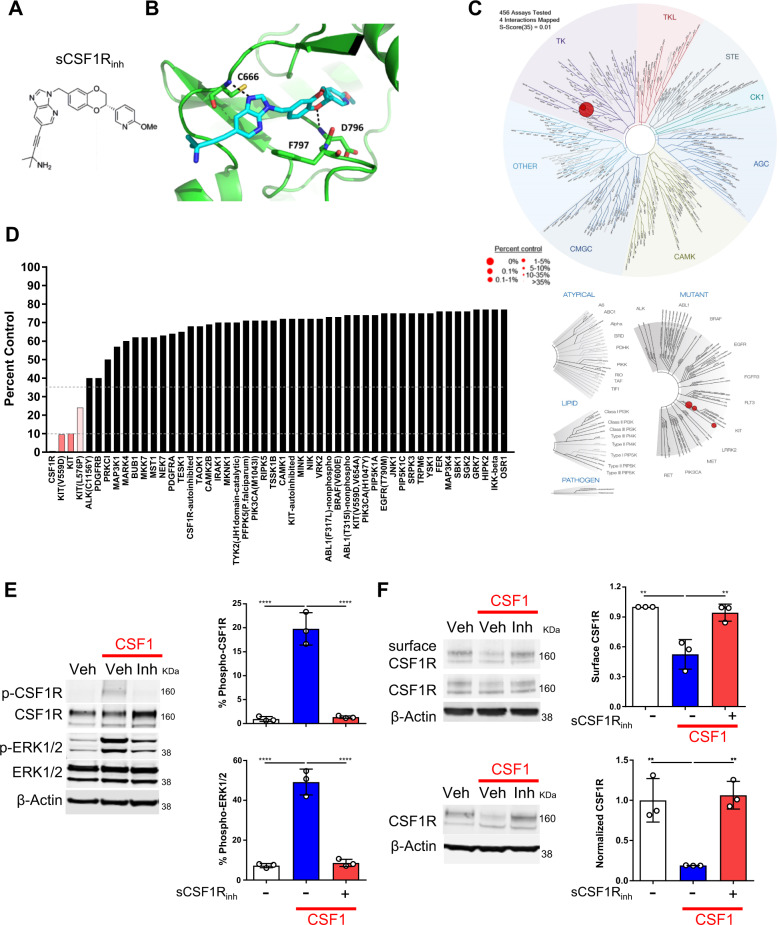
Generation and validation of sCSF1R_inh_, a novel small molecule CSF1R inhibitor. **a** The chemical structure of the CSF1R inhibitor, ((*S*)-4-(3-((2-(6-methoxypyridin-3-yl)-2,3-dihydrobenzo[*b*][1,4]dioxin-6-yl)methyl)-3*H*-imidazo[4,5-*b*]pyridin-6-yl)-2-methylbut-3-yn-2-amine (sCSF1R_inh_). **b** CSF1R crystal structure in combination with sCSF1R_inh_ shows how the inhibitor binds in a DFG-out conformation and makes key interactions with the hinge region. **c** TREEspot™ interaction map is an artistic representation of the human kinome phylogenetic tree. Kinases found to bind sCSF1R_inh_ are highlighted with red circles where larger circles indicate higher affinity binding. **d** KINOMEscan™ screening platform is an active site-directed competition binding assay to quantify the interaction between sCSF1R_inh_ and 450 human kinases. The percent control score demonstrates that sCSF1R_inh_ is highly selective for CSF1R. **e** CSF1 (100 ng/mL) stimulation of BV2 microglia induces CSF1R and ERK1/2 phosphorylation after 5 min and sCSF1R_inh_ (500 nM) blocks these phosphorylation events (**f**) After 10 min, CSF1 stimulation of BV2 microglia leads to endocytosis of the receptor and sCSF1R_inh_ (500 nM) maintains CSF1R surface expression. Upon endocytosis, CSF1R is trafficked to the lysosome for degradation and sCSF1R_inh_ (500 nM) blocks CSF1R degradation. Data points represent quantification from a single Western Blot. Graphical columns represent the mean and standard deviation of biological triplicates. Statistical significance was determined by a one-way ANOVA and *p* values are indicated by ****p < 0.0001.

To alter the harmful neuroinflammatory response in progressive MS, we generated a small-molecule CSF1R inhibitor with activity against both mouse and human CSF1R. Specifically, we developed (*S*)-4-(3-((2-(6-methoxypyridin-3-yl)-2,3-dihydrobenzo[*b*][1,4] dioxin-6-yl)methyl)-3*H*-imidazo [4,5-*b*]pyridin-6-yl)-2-methylbut-3-yn-2-amine (sCSF1R_inh_, Fig. 3a, b) as a result of a structure-based discovery effort aimed at the identification of potent and selective CSF1R inhibitors^43^. sCSF1R_inh_, is a type II kinase inhibitor, that binds to CSF1R in a DFG-out conformation (Fig. 3b). The compound makes a key hydrogen bond interaction to hinge-region residue Cys-666 through N-3 of the imidazo[4,5-*b*]pyridine ring. The benzodioxane moiety occupies an allosteric pocket adjacent to the ATP binding site, and a second key hydrogen bond interaction is made between O-1 of the benzodioxane ring and Asp-796 of the DFG motif. The alkynylamine function located on the imidazo[4,5-*b*]pyridine ring extends from the ATP pocket into solvent-exposed space. The binding specificity of sCSF1R_inh_ was assessed in an active site-directed competition binding assay (KINOMEscan) to measure the interactions between sCSF1R_inh_ and 450 human kinases and disease-relevant mutant variants. sCSF1R_inh_ is highly selective for CSF1R as compared to other kinases with a selectivity score of S(35) = 0.005 (Fig. 3c, d). Furthermore, sCSF1R_inh_ exhibited excellent pharmacokinetic properties including good oral bioavailability and CNS penetrance (Supplementary Fig. 3A), which makes it amenable for both in vitro and in vivo experiments.

### sCSF1R_inh_ blocks CSF1R signaling in vitro

Upon CSF1 ligand binding, CSF1R dimerizes and undergoes receptor auto-phosphorylation. This phosphorylation event initiates the CSF1R signaling cascade and leads to the phosphorylation of other downstream molecules like ERK1/2 and eventually to the endocytosis and degradation of CSF1R via the lysosomal pathway^44^. To assess the impact of sCSF1R_inh_ on the CSF1R signaling cascade, we stimulated BV2 microglia with CSF1 and assessed CSF1R signaling via western blot analysis. A short pulse of CSF1 stimulation induced robust CSF1R and ERK1/2 phosphorylation, which was inhibited in the presence of the sCSF1R_inh_ (Fig. 3e). Longer exposure to CSF1 stimulation leads to the internalization of the receptor and subsequent degradation of CSF1R, and sCSF1R_inh_ also abrogated this process (Fig. 3f). We observed similar findings in murine primary microglia and bone marrow-derived macrophages (data not shown) as well as with IL-34 stimulation (Supplementary Fig. 4). Together these data not only show how the kinase activity of CSF1R regulates both its own internalization and degradation, but also demonstrate that sCSF1R_inh_ effectively inhibits CSF1R signal transduction downstream of the receptor.

### sCSF1R_inh_ attenuates CSF1-induced proliferation and cytokine production

**Fig. 4:**
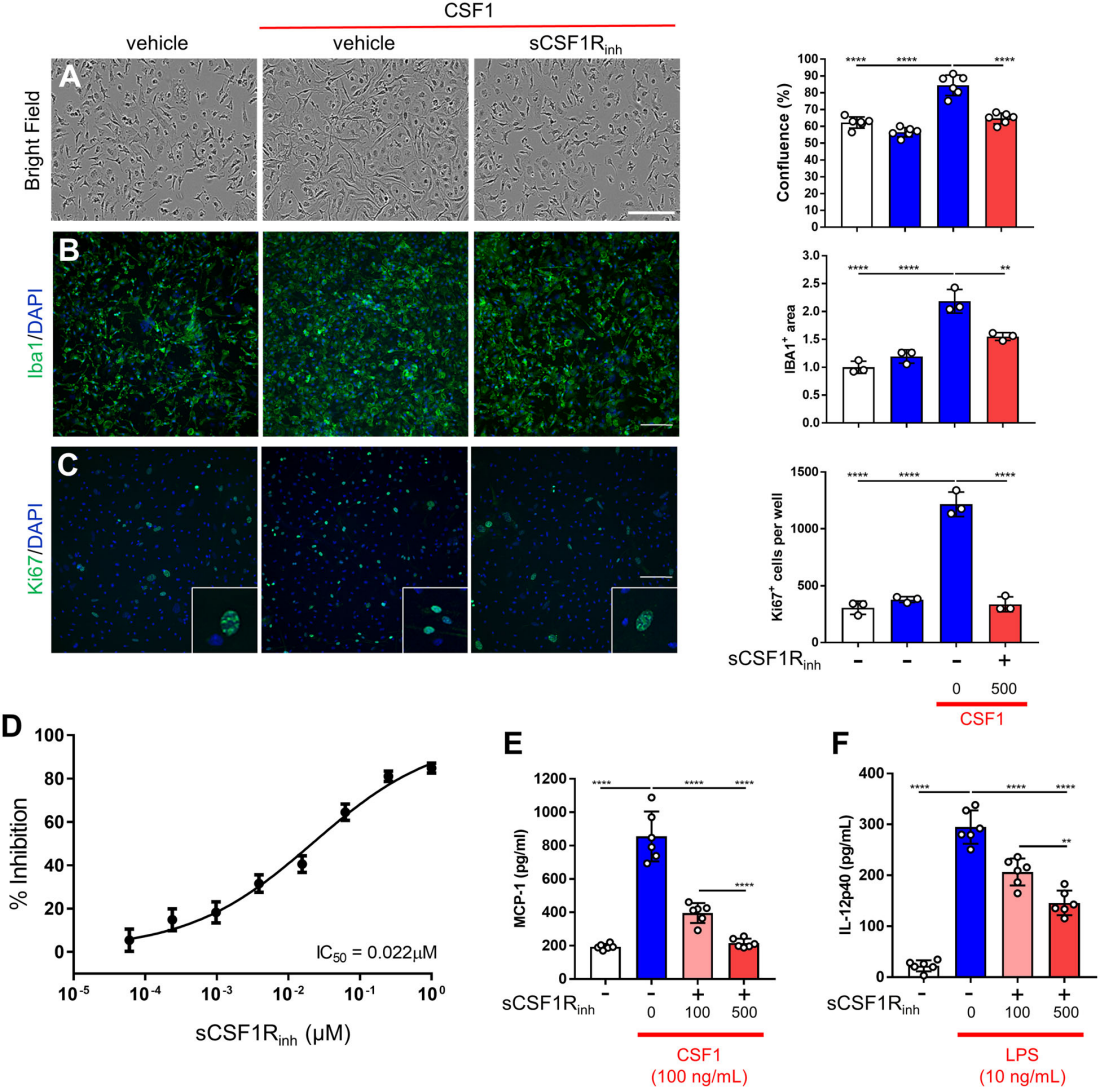
sCSF1R_inh_ blocks murine microglia proliferation and attenuates pro-inflammatory signaling. **a** Bright field images illustrate the impact of CSF1 stimulation on cell confluence in primary murine microglia cultures. Quantification with IncuCyte Zoom software demonstrates that sCSF1R_inh_ blocks CSF1-induced increases in cell coverage. Data points represent the average cell coverage per well and graphical columns represent the mean and standard deviation (n=6). Statistical significance was determined by a one-way ANOVA and p values are indicated by ****p < 0.0001. **b** Immunocytochemistry with an Iba1 antibody shows microglial morphology and number following CSF1 stimulation. Quantification with IncuCyte Zoom software further confirms that sCSF1R_inh_ abrogates CSF1-induced increases in Iba1 coverage. Data points represent the average Iba1 area per well and graphical columns represent the mean and standard deviation (n=3). Statistical significance was determined by a one-way ANOVA and *p* values are indicated by **p < 0.01 and ****p < 0.0001. **c** Ki67 immunostaining was utilized to visualize microglial proliferation following CSF1 stimulation. Ki67^+^ cells were quantified with IncuCyte Zoom software. Data points represent the average number of Ki67^+^ cells a per well (from nine images) and graphical columns represent the mean and standard deviation (*n* = 3). Statistical significance was determined by a one-way ANOVA and *p* values are indicated by ****p < 0.0001. **d** CSF1 (5 ng/mL) induced murine macrophage proliferation over the course of 24 h as measured by H3 incorporation and sCSF1R_inh_ blocked proliferation in a concentration-dependent manner. Data points and error bars represent the mean and standard deviation (*n* = 9); the IC_50_ was calculated with Prism 6 (GraphPad Software). **e** CSF1 stimulation (100 ng/mL) induced MCP-1 release from primary murine microglia and sCSF1R_inh_ blocked MCP-1 production in a concentration-dependent manner (**f**) LPS (10 ng/mL) induced a significant increase in IL-12p40 production by primary murine microglia and sCSF1R_inh_ significantly reduced IL-12p40 in a concentration-dependent manner. Data points represent the protein concentration per well and graphical columns represent the mean and standard deviation. Statistical significance was determined by a one-way ANOVA and *p* values are indicated by **p < 0.01, ****p < 0.0001.

A key observation from post-mortem MS tissue is that the CSF1-CSF1R axis is markedly elevated in progressive MS, and this elevation correlates with increased markers of proliferation. To assess the impact of sCSF1R_inh_ on the downstream cellular readouts of CSF1R signaling, we stimulated primary murine microglia with CSF1 and examined the impact on cellular morphology and confluence via live imaging. Specifically, CSF1 induced an increase in cellular confluence and this effect was blocked by sCSF1R_inh_ (Fig. 4a). This increase was also observed via microglial immunocytochemistry and high content imaging (Fig. 4b). To assess whether the CSF1-induced change in cellular confluence was mediated by proliferation or a shift in morphology, we performed immunocytochemistry with Ki67. CSF1 stimulation produced a marked increase in the number of Ki67^+^ cells per well and sCSF1R_inh_ completely abolished this effect (Fig. 4c). These data demonstrate that CSF1 induces microglial proliferation and sCSF1R_inh_ blocks this function. In MS disease progression, there is a marked recruitment of macrophages into the CNS as seen by histopathological analysis of MS patient tissue as well as in preclinical MS models^7^. To further examine the effect of CSF1R inhibition on macrophage proliferation, we stimulated bone marrow-derived macrophages with CSF1 and assessed cell proliferation by measuring the incorporation of tritiated thymidine. As with microglia, CSF1 stimulation-induced proliferation in these murine macrophages and sCSF1R_inh_ treatment significantly decreased proliferation in a concentration-dependent manner (Fig. 4d**)**. We generated a concentration–response curve for the effect of sCSF1R_inh_ and determined the IC_50_ value to be 22 nM for the inhibition of murine bone marrow-derived macrophage proliferation.

To further investigate the functional consequence of CSF1R inhibition, we evaluated the impact of sCSF1R_inh_ on pro-inflammatory signaling in primary murine microglia by stimulating cells with CSF1 or LPS and examining chemokine/cytokine production by ELISA. CSF1 stimulation-induced MCP-1 release from primary murine microglia and sCSF1R_inh_ blocked MCP-1 production in a concentration-dependent manner (Fig. 4e). Similarly, LPS stimulation induced an increase in IL-12p40 release and sCSF1R_inh_ significantly reduced IL-12p40 in a concentration-dependent manner (Fig. 4f). Importantly, neither of these stimulation paradigms induced a change in cell number demonstrating that CSF1R inhibition impacts cytokine/chemokine production independent of cell proliferation. Collectively, the data demonstrate that CSF1R inhibition reduces deleterious microglia/macrophage proliferation and has the potential to attenuate the pro-inflammatory phenotype of these cells.

### sCSF1R_inh_ reduces microglial survival in a context-dependent manner

**Fig. 5:**
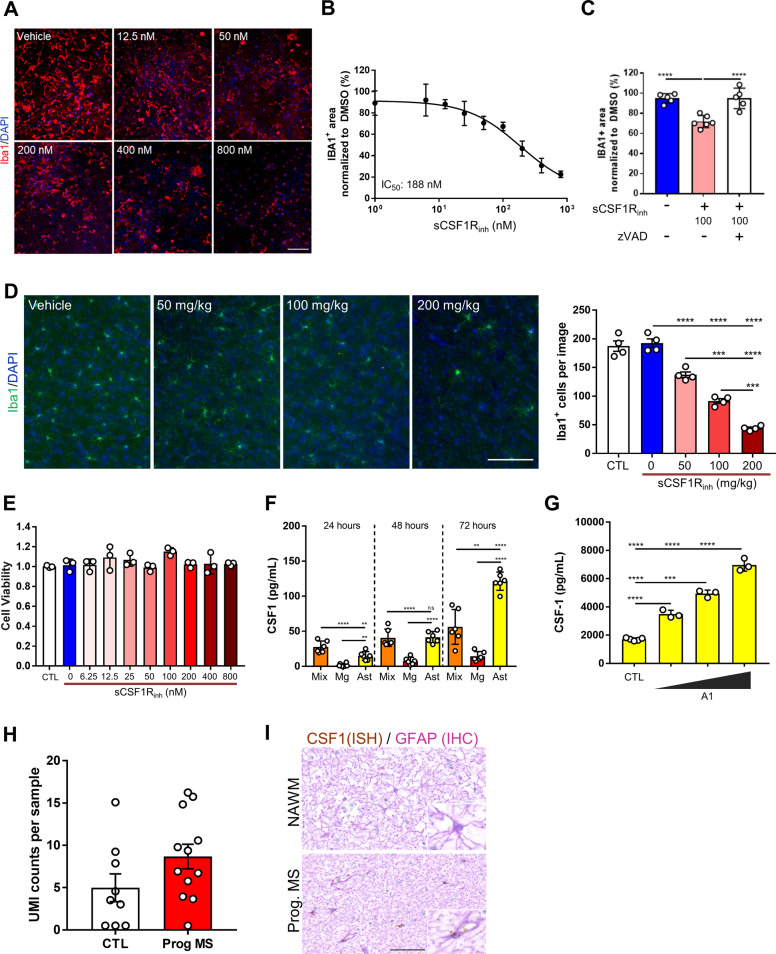
CSF1R inhibition regulates microglial cell turnover. **a** Murine mixed glial cultures were treated with sCSF1R_inh_ and Iba1 immunocytochemistry was used to identify microglia after 3 days. Representative images from the INCell Analyzer Imaging system demonstrate a qualitative reduction in microglia following CSF1R inhibition. Scale bar: 100 µm. **b** Quantitative analysis of Iba1^+^ area reveals that sCSF1R_inh_ significantly depletes microglia in a concentration-dependent manner. Data points represent the mean and standard deviation (*n* = 3); the IC_50_ was calculated with Prism 6 (GraphPad Software). **c** Murine mixed glial cultures pretreated with zVAD blocks sCSF1R_inh_ induced microglial cell death after 3 days. **d** Iba1 immunohistochemistry reveals a dose dependent decrease in the number of cortical microglia in naïve C57BL/6 mice treated with vehicle versus sCSF1R_inh_ for 7 days. Scale bar: 100 µm. Manual quantification of Iba1^+^ cells confirmed these qualitative observations. Data points represent the average Iba^+^ cell count per animal (*n* = 3 images/animal, *n* = 4 animals/group). Graphical columns represent the mean and standard error. Statistical significance was determined by a one-way ANOVA and *p* values are indicated by ***p < 0.001, ****p < 0.0001. **e** Primary murine microglia were treated with sCSF1R_inh_ and cell viability was assessed after 5 days utilizing Promega’s Cell Titer Glo 2.0 Assay Kit. sCSF1R_inh_ treatment had no impact on microglial cell survival at the concentrations assessed in this experiment. Data points represent the viability in each well and graphical columns represent the mean and standard deviation of three wells. **f** CSF1 levels were elevated in conditioned media from mixed murine glial cultures as well as primary microglia and astrocytes. Data points represent the protein concentration per well and graphical columns represent the mean and standard deviation of six wells. Statistical significance was determined by a one-way ANOVA performed and *p* values are indicated by **p < 0.01, ****p < 0.0001. **g** The A1 cocktail induced a concentration-dependent increase in CSF1 production by primary human astrocytes. Data points represent the protein concentration per well and graphical columns represent the mean and standard deviation of six wells. Statistical significance was determined by a one-way ANOVA performed and *p* values are indicated by **p < 0.01, ****p < 0.0001. **h** Pseudo bulk sequencing analysis of CSF1 expression in astrocytes from control versus MS patient from previously published CNS single nuclei sequencing data^46^. **i** In situ hybridization with CSF1 probe and immunohistochemistry with a GFAP antibody reveals the presence of CSF1 transcripts within astrocytes in the progressive MS brain. Scale bar: 50 µm.

CSF1R inhibition alters cellular proliferation in a concentration-dependent manner. Previous reports have also demonstrated that CSF1R signaling is required for microglial survival and that CSF1R inhibition depletes microglia in vivo^15^. The dual role of CSF1R in both these pathways suggests that this receptor regulates the balance between proliferation and survival. To test the impact of sCSF1R_inh_ on CSF1-mediated microglial survival, we used sCSF1R_inh_ in a murine mixed glial culture which contains microglia as well as astrocytes, oligodendrocytes, and endothelial cells. In this culture system, we found that Iba1^+^ microglia are highly susceptible to sCSF1R_inh_ and undergo cell death in a time and concentration-dependent manner (Fig. 5a, b). After three days of treatment, we determined the IC_50_ value of sCSF1R_inh_ to be 188 nM for the depletion of murine microglia (Fig. 5b). Moreover, microglial depletion in the mixed glial system was caspase dependent, as pre-treatment with the pan-caspase inhibitor, zVAD (20µM) blocked sCSF1R_inh_ induced cell death (Fig. 5c).

This CNS-penetrant inhibitor was also able to alter microglial homeostasis in vivo. sCSF1R_inh_ treatment in naïve C57BL/6 mice led to an exposure dependent decrease in the number of microglia in the CNS as quantified by Iba1 immunohistochemistry (Fig. 5d, Supplementary Fig. 5E). These data demonstrate that sCSF1R_inh_ modulates microglial survival in vivo, and that microglial function in the mixed culture system mimics some of the key aspects of the in vivo experiment. Interestingly, sCSF1R_inh_ did not impact cellular survival in primary murine microglial cultures even at high concentrations (800 nM) for 5 days (Fig. 5e). This result is also replicated with another CSF1R inhibitor, PLX3397 (Supplementary Fig. 5E). One potential explanation for this dichotomy is that primary microglia express low levels of the receptor in this pure culture system. However, primary murine microglia cultures expressed CSF1R and responded to CSF1 stimulation as expected, suggesting that the inability of sCSF1R_inh_ to induce cell loss is not related to CSF1R itself (*data not shown*, Fig. 4). Alternatively, this discrepancy may be due to other CNS resident cells within the mixed culture system that provide signals to sensitize microglia to CSF1R-dependent cell death. Consistent with this hypothesis, we found that CSF1 levels were markedly higher in mixed glial cultures as compared to primary microglia (Fig. 5f). We also observed similarly high levels of CSF1 in pure murine astrocyte cultures (Fig. 5f) as well as primary human astrocytes (Fig. 5g). A recent study suggests that microglial-astrocyte communication drives neuroinflammation and toxicity in the CNS^45^. Interestingly, CSF1 in human astrocytes was robustly induced in the presence of A1 stimulation (IL-1α, TNF, and C1q, Fig. 5g). These findings suggest that astrocyte-derived CSF1 may lead to an altered microglial phenotype that confers CSF1-dependence on microglial survival. Using previously published single nuclei RNA-sequencing data from control versus MS patients^46^, we performed a pseudo bulk sequencing analysis of CSF1 expression in astrocytes. CSF1 expression levels were relatively high compared to other cell types and expression levels trended up in MS patients (Fig. 5h). Using histology to specifically examine progressive disease, we observed increased staining for CSF1 mRNA in astrocytes from progressive MS patients (Fig. 5i), suggesting that astrocytes may be driving microglial signaling through CSF1R.

### sCSF1R_inh_ reduces inflammatory microglial proliferation in vivo

CSF1R signaling appears to be a critical node for modulating microglia and macrophage function in response to inflammatory-driven stimuli. To test how sCSF1R_inh_ impacts cellular responses in vivo, we first utilized an acute LPS model to induce microglial activation in the CNS^47^. In this model, a low dose of LPS (1 mg/kg) was administered by intraperitoneal injection daily for 4 days and on the 5th day microglia and infiltrating macrophages in the CNS were quantified by flow cytometry (Supplementary Fig. 6). With LPS stimulation alone, we observed a significant increase in the number of CD11b^+^CD45^+^ microglia and infiltrating macrophages in the CNS (Supplementary Fig. 7A–C). sCSF1R_inh_ was administered just prior to LPS injection each day and after five days mononuclear phagocytes were isolated for flow cytometry analysis. We found that sCSF1R_inh_ significantly reduced the number of microglia and infiltrating macrophages in a concentration-dependent manner (Supplementary Figs. 7A–C, 3B). To elucidate the mechanism of CSF1R inhibition in this model, we performed the LPS acute model with BrdU administration to quantify cell proliferation. sCSF1R_inh_ significantly reduced the percent of BrdU^+^ microglia but had no impact on BrdU uptake in infiltrating macrophages (Supplementary Fig. 7D–F). These findings suggest that sCSF1R_inh_ blocks microglial proliferation in the context of neuroinflammation but reduces the number of infiltrating macrophages through a different mechanism of action in this model.

### sCSF1R_inh_ attenuates chronic neuroinflammation

**Fig. 6:**
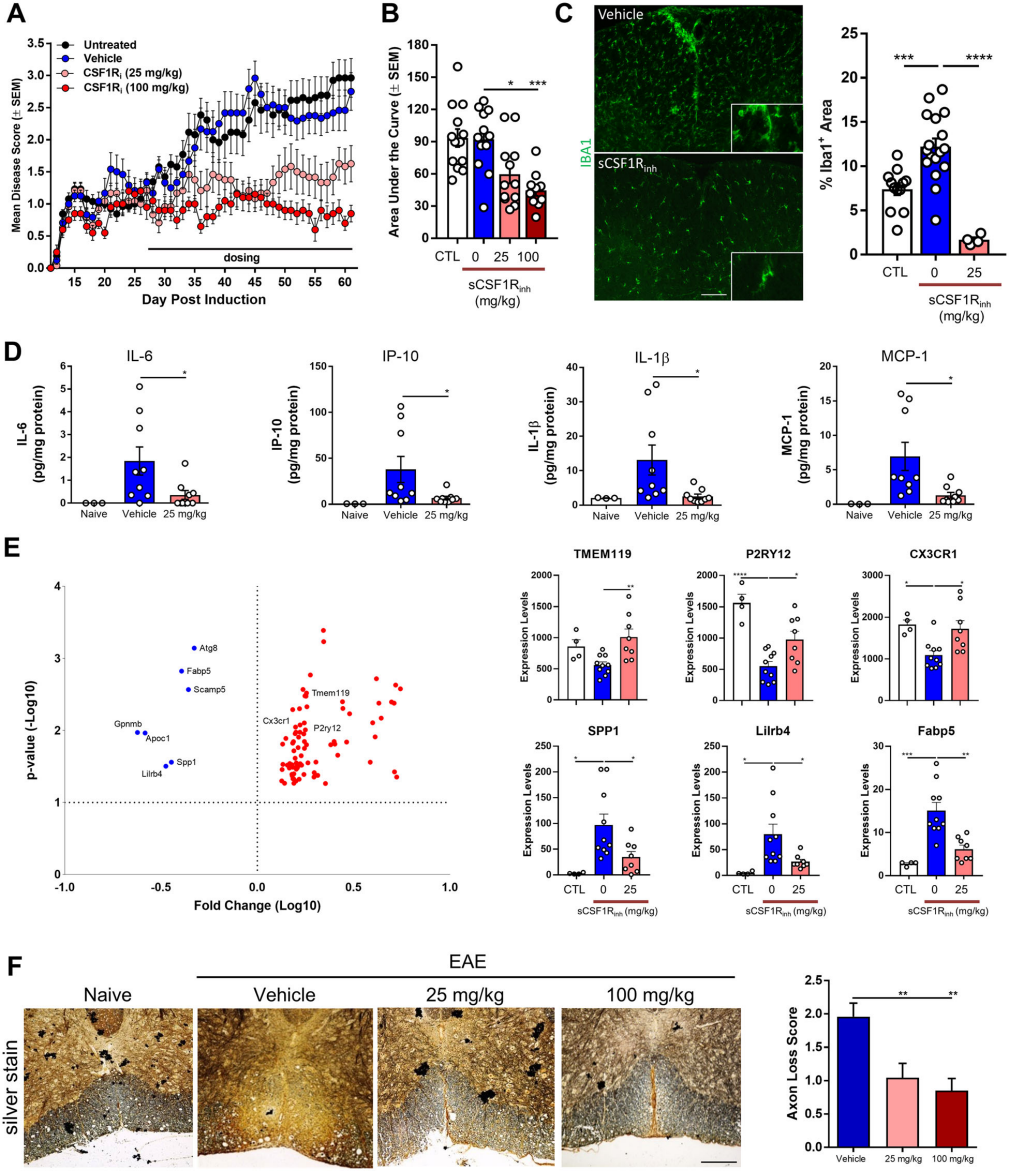
sCSF1R_inh_ significantly suppresses clinical disease progression in the NOD-EAE model. **a** Disease scores demonstrate the initial relapsing/remitting and progressive stages of the MOG-induced NOD-EAE model. Upon treatment with sCSF1R_inh_ at Day 28, mean disease scores were significantly reduced. Data points represent the mean and standard error of the mean (*n* = 12–13). A two-way ANOVA was used to determine the statistical significance of the differences between vehicle and sCSF1R_inh_ treated groups. For sCSF1R_inh_ at 25 mg/kg, *p* ≤ 0.05 on Day 36–37, 39–50, 52–61 while sCSF1R_inh_ at 100 mg/kg had *p* ≤ 0.05 on Day 35–61. **b** Area under the curve was calculated for the clinical disease course and sCSF1R_inh_ significantly reduces paralytic symptoms at both doses. Data points represent the area under the curve for each animal and graphical columns represent the mean and standard error. Statistical significance was determined by a one-way ANOVA and *p* values are indicated by *p < 0.05, ***p < 0.001. **c** Iba1 immunohistochemistry was used to visualize microglia and infiltrating macrophages in the spinal cord and sCSF1R_inh_ significantly reduced the Iba1^+^ area. Data points represent the Iba1^+^ area for each animal while graphical columns represent the mean and standard error. Statistical significance was determined by a one-way ANOVA and *p* values are indicated by ***p < 0.001, ****p < 0.0001. **d** Inflammatory cytokine production in the spinal cord was assessed with ELISA and sCSF1R_inh_ treatment at 25 mg/kg significantly reduced IL-6, IP-10, IL-1β, and MCP-1. Data points represent the protein quantification from a single animal. Graphical columns represent the mean and standard error (*n* = 3, 9, and 8, respectively). A one-way ANOVA was performed to determine statistical significance and *p* values are indicated by *p < 0.05. **e** Quantitative NanoString nCounter mRNA analysis of microglial gene expression in the spinal cord of the NOD-EAE model. sCSF1R_inh_ treatment at 25 mg/kg significantly increased the expression of homeostatic genes such as TMEM119, P2RY12, and CX3CR1 while also reducing the expression of pro-inflammatory genes such as SPP1, Lilrb4, and Fabp5. Data points represent the gene expression for each animal while graphical columns represent the mean and standard error. Statistical significance was determined by a one-way ANOVA and *p* values are indicated by *p < 0.05, **p < 0.01, ***p < 0.001, ****p < 0.0001. **f** Bielschowsky’s silver staining reveals a decrease in axons within the spinal cord of NOD-EAE mice and sCSF1R_inh_ significantly reduced the axonal loss pathology score demonstrating neuroprotection in this model. Statistical significance was determined by a one-way ANOVA and *p* values are indicated by **p < 0.01. Scale bar: 200 µm.

To examine the consequence of CSF1R inhibition in a preclinical MS model, we utilized the C57BL/6 EAE model. In this model, we treated animals at the first sign of clinical symptoms with either vehicle or sCSF1R_inh_ and followed disease progression for fourteen days. Remarkably, therapeutic administration of sCSF1R_inh_ rapidly reduced clinical disease scores after only two days of treatment (Supplementary Fig. 8). To further understand how sCSF1R_inh_ may provide benefit in the context of chronic neuroinflammation, we utilized the NOD-EAE model of progressive MS previously characterized in Fig. 2. As animals entered the progressive stage of the model, they were randomized into either vehicle or sCSF1R_inh_ treatment groups. With this therapeutic dosing regime, sCSF1R_inh_ significantly reduced clinical disease severity as assessed by mean and cumulative disease score (Fig. 6a, b). Microglia and infiltrating macrophages were quantified by Iba1 immunohistochemistry, and sCSF1R_inh_ significantly reduced the presence of these cells within the spinal cord (Fig. 6c).

Based upon our in vitro work, we hypothesized that sCSF1R_inh_ not only attenuates microglia/macrophage proliferation but may also modulate the pro-inflammatory phenotype of these cells within the CNS. To test this hypothesis in vivo, we examined cytokine and chemokine expression in spinal cord homogenate via ELISA. Even at a low dose, sCSF1R_inh_ significantly reduced IL-6, IP-10, IL-1β, and MCP-1 protein expression (Fig. 6d, Supplementary Fig. 9A). These data indicate that the inflammatory profile is altered in the spinal cord of EAE mice treated with sCSF1R_inh_. One possible explanation for this result is that sCSF1R_inh_ simply decreased the number of microglia and infiltrating macrophages and thereby reduced the chemokine/cytokine levels in the CNS. However, based upon our in vitro findings (Fig. 4e), sCSF1R_inh_ may also induce a phenotypic change in microglia and macrophages. To address this question, we purified microglia from the NOD-EAE spinal cord using FACS sorting and assessed gene expression with a quantitative Nanostring nCounter chip containing 400 microglial enriched genes and 150 inflammation-related genes with 6 housekeeping genes^20,48^. Through this analysis, we found 7 genes that were highly upregulated in microglia from NOD-EAE mice and subsequently downregulated with sCSF1R_inh_ treatment (Fig. 6e, Supplementary Fig. 9B). Microglia during neurodegeneration (MGnD) present a specific phenotype which is transcriptionally driven by APOE-TREM2 pathway and is characterized by the expression of genes including *Apoe*, *Trem2*, *Spp1*, *Gpnmb, Fabp5*^20^. The genes that are downregulated after CSF1R inhibition are part of the MGnD signature in microglia. In contrast, we found 82 genes that were highly downregulated in microglia from NOD-EAE and subsequently upregulated with sCSF1R_inh_ treatment (Fig. 6e, Sup plementary Fig. 9B). Interestingly, these genes represented microglial homeostatic genes such as *P2ry12*, *Tmem119*, *Cx3cr1*, *IL34* that are restored following CSF1R inhibition. Overall, we observed a reduction in the expression of the MGnD profile in microglia and a restoration of the homeostatic phenotype following CSF1R pathway inhibition. These data suggest that CSF1R signaling regulates multiple aspects of microglial function, including disease-associated phenotypes. While previous studies have focused on microglial depletion following CSF1R inhibition, our data clearly show that signaling downstream of this receptor can be beneficially modulated in the context of CNS injury.

Our data suggest that CSF1R inhibition can alter both microglial proliferation and deleterious function. To address whether sCSF1R_inh_ also alters neurodegeneration, we performed histopathological assessment of spinal cord tissue sections in the NOD-EAE model. In particular, we utilized Bielschowsky’s silver staining to label neuronal projections and assess axonal loss. We observed a reduction in silver staining in the spinal cord of NOD-EAE mice indicating the presence of axonal loss in this model of progressive MS (Fig. 6f). Animals treated with a low dose of sCSF1R_inh_ appeared to have more silver staining than their vehicle-treated counterparts, suggesting that CSF1R inhibition reduced axonal degeneration. Histopathology scoring was used to quantify axonal loss and sCSF1R_inh_ exhibited a significant neuroprotective effect in vivo (Fig. 6f**)**. In summary, sCSF1R_inh_ treatment not only suppressed neuroinflammation by reducing the proliferation and activation of microglia, but also provided neuroprotection in a preclinical model of progressive disease.

## Discussion

Microglial-dependent neuroinflammation is a key driver of disease progression in MS^49^. In this study, we identified elevated levels of CSF1R and one of its ligands, CSF1, in the CNS of progressive MS patients. These data suggest that the CSF1R signaling node is activated in MS and may drive deleterious neuroinflammation particularly during disease progression. Similar to our findings from post-mortem progressive MS samples, a CSF1R-dependent transcriptional node was also identified in the murine NOD-EAE model. This signaling node was positively correlated with the severity of paralytic symptoms in this model of progressive disease. To interrogate the role of CSF1R in this MS mouse model as well as in macrophage and microglia biology, we developed a novel kinase inhibitor that is potent, selective, and amenable to in vivo experiments. We utilized this small molecule to demonstrate that inhibition of CSF1R signaling attenuates inflammatory signaling, reduces peripheral immune infiltration into the CNS, and ameliorates EAE disease severity. Importantly, we used a therapeutic dosing paradigm by initiating sCSF1R_inh_ treatment after the onset of disease and observed robust efficacy, suggesting that this strategy could be translationally relevant. These data also highlight the key role of the innate immune system in mediating CNS damage in the context of autoimmunity and the importance of targeting macrophages and microglia to potentially prevent disease progression.

Many studies have interrogated the role of microglial cells in inflammatory diseases of the CNS using a depleting dose of a CSF1R inhibitor. In particular, studies have utilized both the C57BL/6 EAE and cuprizone models to understand the role of microglia and infiltrating macrophages in MS^22,29,50^. Some studies have shown marked improvement in EAE disease score in the presence of a CSF1R inhibitor^29^ as well as a blockade of demyelination in the cuprizone model^50^. Meanwhile, other studies have identified deficits in myelin clearance^22^. These data suggest that depletion of microglial cells in the CNS may negatively impact some regenerative functions in disease. In particular, microglial depletion may alter myelin clearance after injury, which may be harmful in the context of demyelinating diseases such as MS. Our work takes a more nuanced approach in understanding the role of CSF1R in the context of neuroinflammation. Importantly, the CSF1-CSF1R axis is not only critical for microglial survival, but also regulates the proliferation and molecular phenotype of these innate immune cells. Using sCSF1R_inh_, we interrogated the dynamics of these three distinct cellular readouts of CSF1R signaling both in vitro and in vivo.

First, we confirmed that CSF1R signaling regulates microglial survival and our in vitro data suggests that this signaling node is normally regulated in a non-cell autonomous fashion. In particular, we identified astrocytes as a major source of CSF1 in our in vitro studies and confirmed this expression pattern in progressive MS patient samples. These data are consistent with previous work suggesting that CSF1-CSF1R signaling represents a key response to injury both in the context of demyelination^33^ as well as neuropathic pain^51,52^. One clear mechanism in this injury response is the upregulation of astrocytic CSF1 expression which subsequently functions in a non-cell autonomous manner to modulate the CSF1R-mediated microglial response during neuroinflammation. Our in vitro data demonstrate that astrocytes directly regulate the mode of microglial response to CSF1R inhibition with microglial survival signaling relying on the presence of astrocytes in the cell culture system. In addition, our findings established a concentration-dependent effect of CSF1R inhibition on functionally distinct CSF1R signaling readouts, such as proliferation, survival, and inflammatory signaling. These data suggest that CSF1R signaling regulates microglial function in a context-specific manner, that can also be modulated non-cell autonomously.

In this study, we defined an efficacious CNS exposure of sCSF1R_inh_ that does not lead to the complete depletion of microglia, but rather attenuates their proliferation and neurodegenerative phenotype. In an acute LPS driven CNS inflammation model, we identified CSF1R-dependent microglial proliferation as a potential deleterious neuroinflammatory mechanism. Using post-mortem MS tissue, we also detected higher levels of proliferation in and around white-matter lesions that positively correlated with elevated CSF1R and CSF1 in this region. Our data suggest that increased microglial proliferation may be a driver of CNS inflammation and could contribute to CNS injury and disease progression. Thus, modulating this aspect of microglial function through CSF1R inhibition may be beneficial in halting progression in MS.

This notion is consistent with our findings in the NOD-EAE model of progressive MS, in which attenuating CSF1R signaling in microglia and macrophages not only reduces their proliferative capacity, but also diminishes their neurodegenerative pro-inflammatory phenotype. Our data clearly indicate that CSF1R inhibition can reduce pro-inflammatory cytokines such as IL-6, IP-10, and IL-1β. Moreover, CSF1R inhibition can lower the expression of the MGnD-associated genes in microglia and restore a more homeostatic phenotype in these innate immune cells. By modulating these two facets of microglial activity, CSF1R inhibition is able to provide neuroprotection and significantly ameliorate clinical symptoms in progressive disease. An intriguing observation from our study is that targeting the innate immune system with CSF1R inhibition can dramatically alter disease pathology in T-cell driven EAE mouse models of MS. These data underscore the importance of macrophages and microglia amplifying peripheral immune signals within the CNS, and suggest that understanding their effector function is critical for therapeutic intervention.

The interplay between microglial proliferation and disease-associated phenotype is an interesting avenue to explore further particularly in the context of MS where these two responses may be coupled. For example, one key change that we observed with CSF1R inhibition was a downregulation of MCP1/CCL2 levels within the spinal cord following treatment. This chemokine normally functions to attract peripheral macrophages into the CNS so a reduction in MCP1/CCL2 may provide a mechanism by which sCSF1R_inh_ effectively blocks peripheral macrophage infiltration. Together, these data suggest that CSF1R may represent a key signaling node by which we can therapeutically modulate the proliferation and molecular identity of disease-associated microglia thereby reducing deleterious neuroinflammation and neurodegeneration in progressive MS.

## Materials and methods

### Human brain processing and sample preparation

Frozen human brain tissue from cortical white matter lesions was obtained from the Human Brain and Spinal Fluid Resource Center (HBSFRC) at University of California Los Angeles (http://brainbank.ucla.edu) (Supplementary Fig. 1). The HBSFRC collects, stores, and distributes the highest quality of pre- and post-mortem tissues, including brain, spinal cord, cerebrospinal fluid (CSF), serum, blood cells, and urine, to be used by scientific investigators of neurological and psychiatric diseases. The HBSFRC is supported by The National Institute of Health (NIH).

### Immunohistochemistry and RNAscope hybridization

Fresh frozen human brain tissue was sectioned at 10 µm with a Microm HM560 cryostat (CTR: *n* = 5; Prog MS: *n* = 5). Slides were stored at −80 °C until use. Immunohistochemistry was performed with the Roche Ventana Medical Systems DISCOVERY ULTRA platform. Briefly, slides were wet loaded into the Ventana and exposed to Ventana CC1 for heat-induced epitope retrieval. After heat treatment, tissues were blocked using the ChromoMap DAB kit (Roche, cat# 05266645001). Primary antibodies (rabbit anti-PLP (Abcam, cat# ab183493; 1:1000), rabbit anti-IBA1 (Wako, cat# 019-19741; 1:1000), rabbit anti-Ki67 (Abcam, cat# ab15580; 1:500), or rabbit anti-GFAP (Biolegend cat# 837504; 1:10,000)) were diluted in antibody diluent (Roche, cat# 251-018) and applied to the slides (100 µL/slide). Slides were incubated at 37 °C for 1 h and amplification was then completed with Omni anti-Rabbit HRP (Roche, cat #05269679001) for 20 min. The signal detection was carried out using DAB (Roche, cat# 05266645001) and counterstaining with hematoxylin II (Roche, cat# 05266726001) and bluing solution (Roche, cat# 05266769001).

In situ hybridization was also performed with the Roche Ventana Medical Systems DISCOVERY ULTRA platform. Briefly, slides were thawed for 2 min and placed in 10% neutral buffered formalin for 24 h. The slide was subsequently rinsed twice in PBS for 5 min and wet loaded into the Ventana. Sections were further permeabilized with cell conditioning for 8 min followed by target retrieval at 97 °C and protease treatment (16 min at 37 °C) (ACD mRNA dewax, cat# 323222; ACD mRNA target retrieval VS universal, cat #323221; ACD mRNA protease, cat #322218). Probes were then hybridized for 2 h at 43 °C followed by RNAscope amplification. The following RNAscope probes were used in this study: RNAscope 2.5 VS Probe-*Hs-DapB* (dihydrodipicolinate reductase; cat. #312039), RNAscope 2.5 VS Probe-*Hs*‐*PPIB* (cat. #313909), RNAscope 2.5 VS Probe-*Hs-CSF1R* (cat. #310819) and RNAscope 2.5 VS Probe-*Hs-CSF1* (cat. # 313009). Final signal detection was carried out using mRNA HRP (ACD RNAscope VS Universal HRP Detection Reagents cat #323210) and counterstaining with hematoxylin II (Roche, cat# 05266726001) and bluing solution (Roche, cat# 05266769001). Finally, slides were removed from the Ventana, dehydrated, and coverslipped with Cytoseal XYL (Thermo Scientific, cat# 8312-4). Slides were dried and then imaged with an Aperio Scanscope AT.

Ki67 quantification was performed using the ImageJ Software. Four images were quantified per subject to determine the number of Ki67^+^ cells per image. Each data point represents the mean per subject and a single line connects NAWM and lesion values from the same subject. A paired t-test was used to determine the statistical significance of differences between NAWM and lesion. Statistical analysis was performed with Prism 6 (GraphPad Software) and the *p* value is 0.0223.

### RNA extraction, cDNA preparation, and qPCR on human samples

RNA isolation from the brain was performed with an RNeasy Lipid Tissue Mini Kit (Qiagen, cat# 74804). Briefly, approximately 50 mg of brain tissue (CTR: *n* = 12; RRMS: *n* = 9; Prog MS: *n* = 20) was homogenized in 1 mL of QIAzol Lysis Reagent with a handheld homogenizer. Tubes were then incubated at room temperature for 5 min. Chloroform (200 µL) was added to each sample, tubes were shaken for 15 s and incubated at room temperature for 3 min. The tubes were centrifuged at 4 °C for 15 min at 8000 *g*. The upper aqueous phase was transferred to a new tube and 1 volume 70% ethanol was added. The samples were vortexed and 700 µL sample was pipetted into a RNeasy Mini spin column placed in a 2 mL processing tube and centrifuged for 15 s at 8000 *g*. The flow through was discarded and the remainder of the sample was placed in the spin column for centrifugation (as described). Seven hundred microliters Buffer RW1 were added into each spin column and the tube was centrifuged for 15 s 8000 *g*. The column was then washed with 500 µL Buffer RPE (centrifuge 15 s at 8000 *g*) and 500 µL Buffer RPE (centrifuge 2 min at 8000 *g*). Finally, the spin column was placed in a fresh epi-tube, 50 µL RNase free water was added to the column and spun for 1 min at 8000 *g*. The RNA quantity was then measured using the Nanodrop. cDNA was prepared with the Quantitect Reverse Transcription Kit (Qiagen, cat# 205313). Briefly, the genomic DNA elimination reaction was prepared on ice with 2 µL gDNA Wipeout Buffer 7x and 12 µL RNA template and then incubated for 2 min at 42 °C. The reverse transcription master mix was prepared on ice with 1 µL Quantiscript Reverse Transcriptase, 4 µL Quantiscript RT Buffer, and 1 µL RT primer mix for each sample. This reverse transcription master mix (6 µL) was then added to each RNA template and incubated for 15 min at 42 °C. Finally, the samples were incubated for 3 min at 95 °C to inactivate the Quantiscript Reverse Transcriptase. The cDNA was stored at −80 °C until use. For qPCR, a master mix solution was prepared with the following volumes: 10 µL Fastlane master mix, 5.3 µL H_2_0, 0.5 µL probe (Thermo Scientific, CSF1R: Hs00911250_m1; CSF1: Hs00174164_m1), 0.2 µL housekeeping probe 1 (Thermo Scientific, RPL37a: Hs01102345_m1). The master mix was aliquoted into a 384 well PCR plate and 2 µL sample was added to each well. Samples were run in triplicate.

CSF1R and CSF1 expression levels were calculated as the fold change compared to control samples and all samples were normalized to a housekeeping gene. Technical triplicates were averaged for each sample. Each data point represents the mean per subject and graphical bars represent the mean of each group with error bars depicting the standard error of the mean. A Kruskal–Wallis nonparametric one-way ANOVA with multiple comparisons was used to determine the statistical significance of differences between CTL, RRMS, and Prog MS samples. Statistical analysis was performed with Prism 6 (GraphPad Software) and the *p* values are 0.0148 for CSF1R and 0.0211 for CSF1.

### Western blot

Post-mortem derived tissue (50 mg, CTR: *n* = 6; RRMS: *n* = 5; Prog MS: *n* = 12) was homogenized and lysed in 1% Triton lysis buffer, and samples were centrifuged (13,000 *g*), the supernatant was stored as the soluble fraction. The pellet was further lysed with radioimmunoprecipitation assay (RIPA) buffer (Thermo Scientific, cat# 89900) and sonicated with a fine tip sonicator. Protein concentration was measured using the Rapid Gold BCA Protein Assay Kit (Pierce, cat# A53225). Prior to loading on a gel for Western blot analysis, equal volume and protein concentration in each samples were diluted in 6M urea in loading buffer.

The electrophoresis chamber was assembled with NUPAGE 4-12% Bis-Tris Midi Gel (Life Technologies, NP0321) and filled with 1x NU PAGE MES SDS Running Buffer (Life Technologies, NP002). The samples were loaded into the gel and 10 µL of Chameleon Duo Pre-stained protein ladder (Li-cor, 928-60000) was used to determine band size. The gel was then run at 130mV for approximately 70 min to ensure that all proteins had traveled into the gel appropriately. The gels were removed from the electrophoresis chamber and the protein was transferred from the gel to a membrane using the iBlot Transfer machine and iBlot gel transfer Stacks PVDF Regular (Life Technologies, 1340109). The membranes were then blocked in Odyssey TBS blocking buffer (Li-cor, 927-50000) containing 0.1% Tween 20 for 1 h at room temperature. Primary antibodies were prepared at the appropriate dilutions in blocking buffer and membranes were incubated overnight at 4 °C. The primary antibodies were rabbit anti-CSF1R (1:1000; Cell Signaling Technologies, Cat# 3152), rabbit anti-phospho-CSF1R (1:1000; Cell Signaling Technologies, Cat# 3155), rabbit α-ERK1/2 (1:1000; Cell Signaling Technologies, Cat# 9102), rabbit α-phospho-ERK1/2 (1:1000; Cell Signaling Technologies, Cat# 9101), mouse α-Beta actin (1:10,000; Sigma, Cat# A5441), and mouse α-Tubulin (1:5,000; Sigma, Cat# 5168). The next day, membranes were washed 3 × 5 min in TBS with 0.1% Tween 20 (0.1% TBST) and incubated in secondary antibodies diluted in 0.1% TBST for 1 h at room temperature. The secondary antibody were IRDye 800CW Donkey anti-Rabbit (1:5000; Li-Cor, Cat# 926-32213) and IRDye 680RD Donkey anti-Mouse (1:5000; Li-Cor, Cat# 926-68072). Membranes were then washed 3 × 5 min in 0.1% TBST and once in TBS. The membranes were then imaged on the Licor Odyssey CLX.

CSF1R protein expression was calculated on the Licor Odyssey CLX. Each data point represents a single subject and graphical bars represent the mean of each group with error bars depicting the standard error of the mean. A Kruskal–Wallis nonparametric one-way ANOVA with multiple comparisons was used to determine the statistical significance of differences between CTL, RRMS, and Prog MS samples. Statistical analysis was performed with Prism 6 (GraphPad Software) and the *p* value is 0.0165.

### Cerebrospinal fluid analysis

Patient-derived cerebrospinal fluid was kindly provided by the Haghikia Lab at Ruhr-University Bochum Germany (CIS: *n* = 5; RRMS: *n* = 66; Prog MS: *n* = 64). Cerebrospinal fluid was assayed with the Human Quantikine CSF1 ELISA kit (R&D Systems, cat# DMC00B) or the Human Quantikine IL-34 ELISA kit (R&D Systems, cat# D3400) per the kit instructions. Each data point represents a single subject and graphical bars represent the mean of each group with error bars depicting the standard error of the mean. A Kruskal–Wallis nonparametric one-way ANOVA with multiple comparisons was used to determine the statistical significance of differences between CTL, RRMS, and Prog MS samples. Statistical analysis was performed with Prism 6 (GraphPad Software) and the *p* value is 0.0005 for CSF1 and 0.2911 for IL34.

### NOD-EAE induction, C57BL/6-EAE induction, and scoring

All animal studies were conducted in compliance with the ethical regulations and full approval of Sanofi’s Institutional Animal Care and Use Committee. For the NOD-EAE, female NOD/ShlTJ mice were immunized with an emulsion of MOG_35-55_ peptide (150 µg/mouse) in complete Freund’s adjuvant (CFA) containing 0.6 mg *Mycobacterium tuberculosis* at 9–11 weeks of age. The emulsion was delivered by two subcutaneous injections to the lower back in a volume of 100 µL per injection site. *Bordetella pertussis toxin* (PTX) was administered via intraperitoneal injection on Day 0 and Day 2 at a dose of 150 ng/animal in 200 µL of PBS. Following EAE induction, the mice were monitored daily for paralytic symptoms and scored for their clinical presentation using a progressive scoring system (Score 0: no disease; Score 1: flaccid tail; Score 2: hindlimb weakness; Score 3: hindlimb paralysis; Score 4: Front limb weakness or partial paralysis; Score 5: death). For efficacy studies, animals were randomized into treatment groups according to disease score at Day 28 (*n* = 26 for the RNA-sequencing study and *n* = 10 (100 mg/kg), 12 (vehicle, 25 mg/kg), and 13 (untreated) for the efficacy study). Treatment groups were blinded for the duration of the study and unblinded following final data analysis.

For the C57BL/6 EAE, female C57BL/6 mice were immunized with an emulsion of MOG_35-55_ peptide (250 µg/mouse) in complete Freund’s adjuvant (CFA) containing 0.6 mg *Mycobacterium tuberculosis* at 9–11 weeks of age. The emulsion was delivered by two subcutaneous injections to the lower back in a volume of 100 µL per injection site. *Bordetella pertussis toxin* (PTX) was administered via intraperitoneal injection on Day 0 and Day 2 at a dose of 200 ng/animal in 200 µL of PBS. Following EAE induction, animals were scored for paralytic symptoms as described above for the NOD-EAE. For efficacy studies, animals were randomized into treatment groups as soon as they exhibited a score of 1 (*n*=13 per group). Treatment groups were blinded for the duration of the study and unblinded following final data analysis.

For all EAE studies, the sample size was chosen based upon previous experience with the EAE models as well as previously published studies in the literature. A power analysis was conducted to confirm the sample size selection. For all EAE clinical disease score graphs, data points represent the mean clinical disease score for each day and error bars represent the standard error of the mean. Prism 6 (GraphPad Software) was used to calculate the area under the curve for each animal and a one-way ANOVA with multiple comparisons was used to determine the statistical significance of differences between treatment groups. In the NOD-EAE efficacy study, p values are 0.0165 for vehicle versus 25 mg/kg and 0.007 for vehicle versus 100 mg/kg.

### Isolation of murine spinal cord microglia for flow cytometry

Spinal cords were collected at sacrifice and kept in RPMI on ice until processing. Tissues were removed from RPMI and diced into ~1mm by 1mm pieces with a razor blade. Two milliliters dissociation media (HBSS+2.5 mg/mL Collagenase D + 1mg/mL DNase) were added, mixed well and incubated on a rocker at 37 ºC for 45 min. Each sample was then transferred to a 15 mL conical tube and triturated with a glass pipette for approximately 1 min. The tissue was washed with PBS and centrifuged for 5 min at 2000 rpm. Ten milliliters of PBS were added to each resulting tissue pellet then filtered through a 70 µm cell strainer. The cell strainers and cell suspensions were washed with PBS and centrifuged at 2000 rpm for 5 min. The supernatants were then discarded, and the cell pellets were resuspended with room temperature 30% Percoll/HBSS (5mL/tube) and transferred to a 15 mL conical tube with glass pipets. The conical tubes were centrifuged at 700 *g* for 10 min at room temperature with the centrifuge brake off. After centrifugation, the myelin layer and Percoll were aspirated, and the resultant cell pellets were resuspended with 1 mL PBS and transferred to clean 15 mL conical tubes with glass pipets. The cells were washed with 15 mL PBS and centrifuged at 2000 rpm for 5 min. The supernatants were removed, and the cells were then transferred to the appropriate wells of a round-bottom 96-well plate for staining.

### Flow cytometry for NOD-EAE

Cell suspensions were incubated with primary antibodies for 10 min at room temperature, protected from light. Cells were stained for the following: CD11b (Biolegend, cat# 108745, 1:100), CD45 (Biolegend, cat# 101243, 1:250). Samples were washed twice with staining buffer (0.2% sodium azide, 1.5% BSA, 5mM EDTA, 5% heat-inactivated Fetal Calf Serum in Dulbecco’s Phosphate Buffered Saline) and then fixed with 0.5% PFA. Data were acquired on a Biorad ZE5 flow cytometer and compensation was performed using UltraComp eBeads (Invitrogen, cat# 01-2222-42).

### Immunostaining of murine tissue sections

Tissue sections were placed in a staining jar and allowed to warm up to ambient temperature. Sections were then rinsed in PBS, washed 5 × 5 min in 0.2% PBT (0.2% Triton X-100 in PBS), blocked with 10% donkey serum/0.2% PBT for 2 h at room temperature (500 µL/section) and incubated in primary antibody (rabbit anti-Iba1, 1:500; Wako, cat# 019-19741) diluted in 10% donkey serum/0.2% PBT overnight at 4 °C (500 µL/section). The next day, sections were washed 5 × 5 min in 0.2% PBT and incubated in secondary antibody (donkey anti-rabbit Alexa Fluoro 488, 1:500; Life Technologies, cat# A21206) diluted in 1% donkey serum/0.2% PBT for 2 h at room temperature (250 µL/slide). Sections were then washed 5 × 5 min in 0.2% PBT, incubated in DAPI (1:10,000 in PBS) for 5 min at room temperature, rinsed in PBS, and coverslipped using ProLong Gold antifade mounting media (Life Technologies, cat# P36935). Slides were imaged by fluorescence microscopy using a Zeiss Imager.Z1 microscope equipped with a Zeiss AxioCam MRm digital camera and Zen Pro 2012 Imaging Software.

Microglia were identified by Iba1^+^ immunoreactivity and Iba1+ area was quantified using the ImageJ Software. At least six images were quantified per animal. Data points represent the Iba1^+^ area for each animal, graphical bars represent the mean per group, and error bars represent the standard error of the mean. An unpaired t-test was used to determine the statistical significance of differences between naïve and NOD-EAE mice. Statistical analysis was performed with Prism 6 (GraphPad Software) and *p* values are 0.0002 (cortex), 0.0037 (cerebellum), and <0.0001 (hippocampus and spinal cord).

### RNA extraction and sequencing of murine tissue

Whole spinal cords from naïve or NOD-EAE animals at different days post induction were used for RNA sequencing. For RNA extraction, samples were first randomized then total RNA was extracted using the Qiagen RNeasy Plus kit with optional on-column DNase digest step (all one batch). RNA quantification and quality assessment were performed using a Nanodrop 8000 and a Tapestation 4200, respectively. RIN values ranged from 7.8 to 10. Total RNA was diluted to 1 ng in 10ul of water then using Oligo(dT) priming from Takara’s SmartSeq v4 Ultra Low Input RNA kit, enriched mRNA was converted to full-length cDNA. cDNA was amplified using 11 PCR cycles then cleaned up using AMPure XP beads. The average size of the full-length cDNA ranged from 2500-2800bp after running the Bioanalyzer 2100. Sequencing libraries were prepped and uniquely indexed using Illumina’s Nextera XT kit without modification to the standard protocol. Average size of libraries ranged from 800-900bp using the Tapestation 4200. Each sample was quantified using Qubit then diluted to 4nM and pooled together. To prepare for sequencing, pooled libraries were first denatured with NaOH then diluted to 1.8pM and finally run on an Illumina NextSeq500 using the High output kit and a 2x76bp paired end run.

CSF1R and CSF1 expression were calculated as the fold change compared to naïve animals. Data point represents the expression level of each animal, graphical bars represent the mean of each group, and error bars depict the standard error of the mean. A one-way ANOVA with multiple comparisons was used to determine the statistical significance of differences between naïve and NOD-EAE samples. Statistical analysis was performed with Prism 6 (GraphPad Software) and the p values are 0.0001 (CSF1R: naïve versus NOD-EAE Day 6, 16, 22, 27, and 37), 0.0023 (CSF1R: naïve versus NOD-EAE Day 65), 0.0001 (CSF1: naïve versus NOD-EAE Day 6, 16, 27, 37, and 65), and 0.0003 (CSF1: naïve versus NOD-EAE Day 22).

### Protein crystal structure determination

The protein construct used for crystallization was based upon a previously published construct^53^ and consisted of residues 588-922 of CSF1R with an N-terminal 6-histidine tag followed by a tobacco etch virus cleavage site and with residues 679–752 replaced by residues 577-597 of FGFR1. Protein was expressed using baculovirus-infected insect cells and purified by nickel affinity chromatography, followed by tag cleavage, removal of impurities and uncleaved CSF1R by a second pass over nickel affinity resin and gel filtration chromatography in 20 mM Hepes, 250 mM NaCl, 2 mM TCEP, final pH 7.5. Protein was concentrated to 8 mg/ml and flash-frozen in liquid nitrogen until use in crystallization experiments.

Co-crystals of CSF1R with 3-(oxazolo[4,5-*c*]pyridin-2-yl)aniline were grown in drops consisting of equal volumes of protein at 8 mg/ml and reservoir solution of 0.1 M MES, pH 6.5, 21.3% polyethylene glycol 3350 and 0.25 M ammonium sulfate by cross-seeding using crystals grown in the same condition in the presence of Cpd 1^54^. All crystallization reagents were from Hampton Research (Aliso Viejo, CA). sCSF1R_inh_ was soaked into these crystals by transferring them for 24 h into reservoir solution with 5 mM sCSF1R_inh_ and subsequently frozen by quick dip in soaking solution supplemented with 20 % (w/v) glycerol.

### Data processing and structure determination

Data were processed using autoproc from GlobalPhasing^55^ which relies on XDS^56^ and Aimless^57^ (3) programs. Molecular replacement was carried out using Phaser^58^ of the CCP4 suite^57^. The structure was refined at 2.62 Å using REFMAC5 of CCP4 suite^59^ followed by manual corrections in COOT^60^ to a final Rfree of 0.260 and Rfactor of 0.178. The structure was inspected and analyzed in COOT and Pymol (The PyMOL Molecular Graphics System, Version 1.7.4 Schrödinger, LLC).

### CSF1R inhibitor kinase specificity

sCSF1R_inh_ was tested in a KINOMEscan^TM^ screening platform which utilizes a proprietary active site-directed competition binding assay to quantitatively measure interactions between sCSF1R_inh_ and 450 human kinases and disease-relevant mutant variants. From this screen, a selectivity score was generated by dividing the number of kinases that sCSF1R_inh_ binds to by the total number of distinct kinases tested. This value is calculated using percent of control from the KINOMEscan^TM^ as a potency threshold. For example, S(35) = (number of non-mutant kinases with percent of control less than 35)/(number of non-mutant kinases tested). TREEspot™ is an artistic representation of the human kinome phylogenetic tree. Kinases found to bind marked with red circles and larger circles indicating higher affinity binding.

### Treatment and stimulation of BV2 murine microglia for CSF1R signaling

BV2 murine microglia (Accegen Biotechnology, cat# ABC-TC212S) were suspended at a concentration of 1 × 10^6^ cells/mL and 1 mL of this cell suspension was added to each well of a 12-well plate. BV2 cells were allowed to rest overnight at 37 °C. The following day, cells were serum starved for 4 h and then treated with either dimethyl sulfoxide (DMSO, 0.1%) or sCSF1_inh_ (500 nM) for 30 min at 37 °C. Cells were then stimulated with 100 ng/mL CSF1 (R&D Systems, cat# 416-ML-050) for the appropriate time period. After stimulation, the culturing media was removed from each well, and cells were resuspended in 200 µL of ice cold lysis buffer prepared with a 10 mL Pierce RIPA Buffer (Thermo Scientific, cat# 89900, lot# RK241845), 1 cOmplete Mini tablet (Roche, cat# 11836153001, lot# 25765800), and 100 µL Phosphatase Inhibitor Cocktail II (Sigma, cat# P5726). The plate was then placed on ice and a cell scraper was used to lift the cells off the plate. The lysate was collected into tubes, sonicated 5 × 1 s, and incubated for 15 min at 4 °C. After lysis, the protein concentration of each sample was determined in duplicate using a Pierce Rapid Gold BCA Protein Assay Kit (Thermo Fisher, cat# A53226). Briefly, 5 µl of sample was added to 200 µL BCA working reagent in a 96-well plate and 10 µL of Bovine Serum Albumin Standards were used to generate a standard curve. Sample absorbance was read at 490 nm using the Flex Station and SoftMax Pro 5.4 software and the sample concentrations were calculated as µg/ml protein. Samples were then diluted in lysis buffer to prepare aliquots containing 16 µg protein in 20 µL total volume and run on western blot as described above.

Within each experiment, three separate wells were treated for each condition. Each stimulation paradigm was repeated three different times using BV2 cells from a distinct cell passage. Protein expression was calculated on the Licor Odyssey CLX. Each data point represents a single sample and graphical bars represent the mean of each group with error bars depicting the standard deviation. A one-way ANOVA with multiple comparisons was used to determine the statistical significance of differences between groups. Statistical analysis was performed with Prism 6 (GraphPad Software). For CSF1 stimulation, the p values are 0.0001 (all comparisons in phospho-CSF1R and phospho-ERK1/2), 0.0031 (CSF1R degradation: unstimulated versus CSF1 stimulation), and 0.0021 (CSF1R degradation: CSF1 stimulation versus sCSF1R_inh_ treatment). For IL-34 stimulation, the p values are 0.0001 (phospho-CSF1R: unstimulated versus IL-34 stimulation), 0.0003 (phospho-CSF1R: IL-34 stimulation versus sCSF1R_inh_ treatment), 0.0018 (phospho-ERK1/2: unstimulated versus IL-34 stimulation), 0.002 (phospho-ERK1/2: IL-34 stimulation versus sCSF1R_inh_ treatment), 0.0015 (CSF1R degradation: unstimulated versus IL-34 stimulation), and 0.0017 (CSF1R degradation: IL34 stimulation versus sCSF1R_inh_ treatment).

### Cell surface biotinylation assay with BV2 murine microglia

BV2 murine microglia (Accegen Biotechnology, cat# ABC-TC212S) were suspended at a concentration of 1 × 10^6^ cells/mL and 4 mL of this cell suspension was added to each T75 flask. BV2 cells were allowed to rest overnight at 37 °C, 5%CO_2_. The following day, cells were serum starved for 4 h and then treated with either dimethyl sulfoxide (DMSO, 0.1%) or sCSF1R_inh_ (500 nM) for 30 min at 37 °C. Cells were then stimulated with 100 ng/mL CSF1 (R&D Systems, cat# 416-ML-050) for 5 min or 10 min. After stimulation, the culturing media was removed from each flask, and cells were quickly rinsed with ice cold PBS. A Pierce Cell Surface Protein Isolation Kit (Thermo Scientific, cat# 89881) was used to biotinylated and isolate cell surface proteins. After the cells were rinsed with ice cold PBS, 10 mL of Sulfo-NHS-SS-Biotin (12mg Sulfo-NHS-SS-biotin dissolved in 48 mL ice cold PBS) was added to each flask and flasks were placed on a rocking platform for 30 min at 4 °C. After biotinylation, 500 µL Quenching Solution was added to each flask to quench the reaction. Cells from each flask were gently scraped into solution and transferred to separate 15 mL conical tubes (note: 3–4 mL of ice-cold TBS was used to rinse the flasks). The cells were then centrifuged at 500 *g* for 3 min at 4 °C and the supernatant was subsequently discarded. Cells were resuspended in ice-cold TBS for an additional wash, centrifuged at 500 *g* for 3 min at 4 °C and then resuspended in 350 µL lysis buffer (10 mL Pierce RIPA Buffer (Thermo Scientific, cat# 89900), 1 cOmplete Mini tablet (Roche, cat# 11836153001), and 100 µL Phosphatase Inhibitor Cocktail II (Sigma, cat# P5726)). The lysates were then transferred into epi-tubes, sonicated (1.5 power, five 1-s bursts), and incubated for 30 min at 4 °C. After lysis, the protein concentration of each sample was determined in duplicate using a Pierce Rapid Gold BCA Protein Assay Kit (Thermo Fisher, cat# A53226) as described above. The sample concentrations were calculated as µg/ml protein. Samples were then diluted in lysis buffer to normalize the protein concentration across all samples and a 30 µL aliquot was generated to serve as a loading control (input sample).

First, NeutrAvidin agarose gel (400 µL) was added to each column and the tubes were centrifuged at 1000 *g* for 1 min. The flow-through was discarded and the gel was washed again with 400 µL wash buffer. The column was centrifuged again at 1000 *g* for 1 min and the flow-through discarded. The bottom cap was then applied to each column and the cell lysate was added to a separate column. The lysate/gel mixture was then incubated for 60 min at room temperature on a rotator. After incubation, the bottom caps were removed and the tubes were centrifuged at 1000 *g* for 1 min. The columns were then washed four times as follows: Add 400 µL wash buffer to each column, invert columns to mix, centrifuged columns at 1000 *g* for 1 min and discard flow through. After the final wash, the protein was eluted from the agarose gel with 150 µL SDS-Page sample buffer containing 50 mM DTT, incubated at room temperature for 60 min. The columns were then placed in a new collection tube and centrifuged at 1000 *g* for 2 min. The eluates were then analyzed in combination with the original input samples via Western Blot.

Within each experiment, three separate wells were treated for each condition. Each stimulation paradigm was repeated three different times using BV2 cells from a distinct cell passage. Protein expression was calculated on the Licor Odyssey CLX. Each data point represents a single sample and graphical bars represent the mean of each group with error bars depicting the standard deviation. A one-way ANOVA with multiple comparisons was used to determine the statistical significance of differences between groups. Statistical analysis was performed with Prism 6 (GraphPad Software). For CSF1 stimulation, the *p* values are 0.0019 (unstimulated versus CSF1 stimulation) and 0.0037 (CSF1 stimulation versus sCSF1R_inh_ treatment).

### Isolation of primary murine microglia for in vitro assays

Brains from four day old C57BL/6 mice were harvested and pooled in DMEM/F12-Glutamax/10%FBS/1%Pen/Strep/100 µM non-essential amino acids/2 mM sodium pyruvate (“complete DMEM/F12”) and kept on ice until processing. Upon arrival in the lab, the brains were transferred into warm 0.25% trypsin (2 mL/brain) and incubated at 37 °C while rotating for 30 min. The dissociation reaction was quenched with an equal volume of complete DMEM/F12. The tissue was centrifuged at 300 *g* for 7 min, and the supernatant was then carefully removed. The tissue pellet was washed 3 times with complete DMEM/F12 and centrifuged at 300 *g* for 7 min. Supernatant was carefully removed after each wash step with a pipet rather than by vacuum aspiration. After the final wash, the tissue was brought up in complete DMEM/F12, slowly triturated until no chunks were visible, and filtered through a 70 µM cell strainer. The resultant single-cell suspension was washed with complete DMEM/F12, centrifuged at 200 *g* for 7 min and resuspended with complete DMEM/F12. The cells were distributed evenly amongst T150 tissue culture flasks (1 flask/mouse), and the final volume was brought up to 35 mL with complete DMEM/F12. The cells were fed with a complete medium change 5, 8, and 11 days later.

On day 12, each flask was washed with 10 mL PBS. Five milliliters 0.25% trypsin were added to each flask, and the flasks were placed on a rocker at room temperature for 15 min. Ten milliliters complete DMEM/F12 were added to each flask, and the cells were gently triturated to break up cell aggregates. The single-cell suspensions were then filtered through 70 µM cell strainers and centrifuged at 200 *g* for 6 min. The cells were then pooled, counted and resuspended at 1 × 10^8^ cells/mL in PBS/2%FBS/1mM EDTA (“separation media”). The cells were transferred to 5mL polystyrene FACS tubes, and the CD11b-PE + FcR block reagent provided in the Mouse CD11b Positive Selection Kit (StemCell cat# 18770) was added to the tubes and incubated at room temperature for 15 min. The PE selection cocktail was then added to the FACS tubes and mixed well with a pipet tip. The sample again incubated for 15 min at room temperature. The EasySep Magnetic Nanoparticles provided in the kit were gently mixed and added to the tubes and incubated at room temperature for 10 min. The tubes were placed in the EasySep Magnets (StemCell cat# 18000) and allowed to sit for 7 min. In one fluid motion, the unlabeled cells in the buffer were poured off while the tube was still in the magnet. The tube was then removed from the magnet, separation media was added to the tube, and the tube was placed back in the magnet for another 7 min. This washing process was done for a total of 4 times to remove all unlabeled cells. After the last wash, the labeled CD11b^+^ cells were resuspended in complete DMEM/F12.

### Cell proliferation assay in primary murine microglia

Primary mouse microglia were plated at 50,000 cells per well in a 96-well plate. Microglia cells were allowed to rest 24 h at 37 °C, 5% CO_2_. The following day, the media was removed and cells were treated with either dimethyl sulfoxide (DMSO) or sCSF1R_inh_ for 30 min at 37 °C, 5% CO_2_. Cells were then stimulated with 100 ng/mL CSF1 (R&D Systems, cat# 416-ML-050) for 24 h—during this time, cells were imaged on the Incu-Cyte Live Imaging System. Cells were then fixed with 4% PFA for 20 min at room temperature. Cells were then rinsed in PBS, washed 3 × 5 min in 0.2% PBT (0.2% Triton X-100 in PBS) and blocked with 10% donkey serum/0.2% PBT for 1 h at room temperature. Cells were then incubated in primary antibody (rabbit anti-Iba1, 1:500; Wako, cat# 019-19741 or rabbit anti-Ki67, 1:500; Abcam, cat# ab15580) diluted in 10% donkey serum/0.2% PBT overnight at 4 °C. The next day, cells were washed 3 × 5 min in 0.2% PBT and incubated in secondary antibody (donkey anti-rabbit Alexa Fluoro 488, 1:500; Life Technologies, cat# A21206) diluted in 1% donkey serum/0.2% PBT for 1 h at room temperature. Cells were then washed 3 × 5 min in 0.2% PBT, incubated in DAPI (1:10,000 in PBS) for 5 min at room temperature, and rinsed in PBS. Plates were then imaged on the IN Cell Analyzer 2200 with nine fields of view acquired per well.

Quantification was performed with the INCell Developer Analysis software, calculating the sum of the area (in μm^2^) of Iba1 staining or the number of Ki67^+^ cells for the nine fields. The average value per field in each well was calculated for each technical triplicate and normalized to the average of the DMSO control wells. Each data point represents a single sample and graphical bars represent the mean of each group with error bars depicting the standard deviation. A one-way ANOVA with multiple comparisons was used to determine the statistical significance of differences between groups. Statistical analysis was performed with Prism 6 (GraphPad Software). For CSF1 stimulation, the p values are 0.0019 (unstimulated versus CSF1 stimulation) and 0.0037 (CSF1 stimulation versus sCSF1R_inh_ treatment). Statistical analysis was performed with Prism 6 (GraphPad Software) and p values are 0.0001 (Confluence: all comparisons, Iba1 area: DMSO + CSF1 versus unstimulated and DMSO, and Ki67: all comparisons) and 0.0013 (Iba1 area: DMSO + CSF1 versus sCSF1R_inh_ treated). Within each experiment, three separate wells were treated for each condition. Each stimulation paradigm was repeated three different times using primary microglial cells obtained from three distinct litters.

### Cell proliferation assay in murine bone marrow macrophages

On day 0, bone marrow cells are collected under sterile conditions and cultured overnight in tissue culture flasks at 1 × 10^6^ cells/mL in DMEM/15%FBS/1%penicillin–streptomycin/1%l–glutamine/50 ng/mL CSF1 (R&D Systems, cat# 416-ML-050). On day 1, the flasks are tapped, and all floating and loosely adherent cells are collected. The cells are counted and resuspended in fresh medium + 50 ng/mL CSF1 at 1 × 10^6^ cell/mL and transferred to 10 cm or 15 cm tissue culture dishes. On days 3 and 6, the media is removed and replaced with fresh medium + 50 ng/mL CSF1. On day 8, adherent cells are washed with PBS, collected, counted and resuspended in fresh medium without CSF1 at 2 × 10^5^ cells/mL. One hundred microliters cells (2 ×10^4^ cells) are then added to the assay wells. After overnight starvation of CSF1, serial dilutions of the sCSF1R_inh_ was prepared and added to the appropriate assay wells. The compound was initially diluted in DMSO to yield serial 4-fold dilutions ranging from 1 mM to 0.46 µM. Each concentration was then diluted 1:167 in media to yield concentrations from 6 to 0.003 µM. Twenty-five microliters of each concentration were then added in triplicate to the assay wells yielding final concentrations of 1 µM to 0.06 nM and 0.1%DMSO. Twenty-five microliters of CSF are also added to each assay well, except the minimum control wells, at a final concentration of 5 ng/mL. At the end of day 9, 1uCi of ^3^H is added to each assay well, and the plates are allowed to incubate at 5%CO_2_ and 37 °C for an additional 16 h. On day 10, cells are harvested onto a filter plate using a 96-well plate harvester then allowed to dry overnight.

Once dry, 25 µL of Beta-Count scintillation fluid is added to each well, and the plate is counted on the Trilux counter using the ^3^H protocol #10. The level of proliferation observed in CSF1-stimulated macrophages was set at 0% inhibition and the level of proliferation observed in unstimulated macrophage proliferation was set at 100% inhibition. Subsequently, the % inhibition generated from each concentration of sCSF1R_inh_ was calculated. Each data point represents the mean % inhibition of each concentration of sCSF1R_inh_ with error bars depicting the standard deviation. The IC_50_ value was calculated with Prism 6 (GraphPad Software). Within each experiment, three separate wells were treated for each condition. Each stimulation paradigm was repeated three different times using primary murine bone marrow macrophages obtained from three distinct animals.

### Cytokine production assay in primary murine microglia

Primary mouse microglia were suspended at a concentration of 5 × 10^5^ cells/mL and 100 µL of this cell suspension was added to each well of a 96-well plate. Microglia cells were allowed to rest overnight at 37 °C, 5% CO_2_. The following day, the media was removed and cells were treated with dimethyl sulfoxide (DMSO), sCSF1R_inh_, or PLX3397 at 37 °C, 5% CO_2_. Cells were then stimulated with 100 ng/mL recombinant mouse CSF (R&D Systems, cat# 416-ML-050) after 30 min pre-treatment or 10 ng/mL lipopolysaccharide (Sigma, cat# L6529) after 24 h pre-treatment. After 24 h of stimulation, the culture supernatant was removed from each well for ELISA. Culture supernatants were assayed with the Mouse Quantikine MCP-1 ELISA kit (cat# MJE00B) and the Mouse Quantikine IL-12p40 ELISA kit (cat# MP400) from R&D Systems per kit instructions. Each data point represents a single well and graphical bars represent the mean of each group with error bars depicting the standard deviation. A one-way ANOVA with multiple comparisons was used to determine the statistical significance of differences between groups. Statistical analysis was performed with Prism 6 (GraphPad Software). For MCP1, the *p* value is 0.0001 for all comparisons. For IL-12p40, the *p* values are 0.0001 for all comparisons to DMSO + LPS and 0.0022 for 100 nM versus 500 nM sCSF1R_inh_ treatment. Within each experiment, six separate wells were treated for each condition. Each stimulation paradigm was repeated three different times using primary microglial cells obtained from three distinct litters.

### Cell survival assays in primary murine mixed glia and primary murine microglial cultures

Mixed glia were seeded at 25,000 cells per well in a PDL-coated black 96-well plate and cultured for 12 days with media changes on DIV5 and DIV8. On day 12, cells were treated with increasing dose of CSF1R inhibitors (sCSF1R_inh_ and PLX3397) for three days. In addition, some plates were pretreated with a pan-caspase inhibitor, zVAD (Enzo, cat# ALX-260-020-M005), at 20 µM 2 h prior to CSF1R inhibitor treatment. After treatment, plates were fixed and stained for CSF1R and IBA1 (as described above). After staining, plates were imaged on the INCell Analyzer 2200 with nine fields of view acquired per well. The quantification was performed on the INCell Developer Analysis software, calculating the sum of the area (in µm^2^) of CSF1R or Iba1 staining for the nine fields. The average area per field in each well was calculated and normalized to the average of the DMSO control wells. Each data point represents the mean % Iba1 area at each concentration of sCSF1R_inh_ with error bars depicting the standard deviation. The IC_50_ value was calculated with Prism 6 (GraphPad Software). Within each experiment, three separate wells were treated for each condition. Each stimulation paradigm was repeated three different times using primary mixed glia obtained from three distinct litters.

Similar experiments were conducted in primary murine microglia and the Cell Titer-Glo Luminescent Cell Viability Assay (Promega, cat# G7570) was used to assay cell survival. Each data point represents a single well and graphical bars represent the mean of each group with error bars depicting the standard deviation. A one-way ANOVA with multiple comparisons was used to determine the statistical significance of differences between groups. Within each experiment, six separate wells were treated for each condition. Each stimulation paradigm was repeated three different times using primary microglia obtained from three distinct litters.

### In vivo microglial depletion study

Adult C57BL/6 female mice (8 weeks old) were orally dosed with sCSF1R_inh_ or PLX3397 for 7 days at 50 mg/kg, 100 mg/kg, and 200 mg/kg (*n* = 4–6 per group). One hour after their last dose, the mice were cardiac perfused with 25 mL of ice cold HBSS. A craniotomy was performed to excise the brain with dissecting tools and the meninges were carefully removed from the surface of the brain. The brain was placed in ice cold 4% PFA overnight at 4 °C and then continuously sectioned in the sagittal plane on a Leica vibratome. Slices were stored at 4 °C until processed for immunohistochemistry.

Quantification was performed using the Analysis tools included in ImageJ Software. Microglia were identified by Iba1^+^ immunoreactivity and manually labeled within the Cell Counter Plugin. At least six images were quantified per animal to determine the number of microglia. Data points represent the Iba1 quantification for each animal, graphical bars represent the mean per group, and error bars represent the standard error of the mean. A one-way ANOVA with multiple comparisons was used to determine the statistical significance of differences between groups. Statistical analysis was performed with Prism 6 (GraphPad Software) and p values are 0.0005 (100 mg/kg versus 200 mg/kg), 0.0007 (50 mg/kg versus 100 mg/kg) and 0.0001 for all other significant comparisons.

### A1 cocktail stimulation of primary human astrocytes

Primary human astrocytes (Gibco cat# N7805-100) were seeded at 20,000 cells per well in a 96 well matrigel-coated plate and maintained in culture for 6 days. On day 7, the media was changed to a serum-free formulation. The following day, cells were stimulated with increasing A1 cocktail concentrations (low = 0.06ng/mL IL-1α + 0.6ng/mL TNF-α + 8ng/mL C1q, med = 0.12ng/mL IL-1α + 1.2ng/mL TNF-α + 16ng/mL C1q, high = 0.3ng/mL IL-1α + 3ng/mL TNF-α + 40ng/mL C1q) for 24 h. Supernatants were collected for quantification of CSF1 with the Human Quantikine CSF1 ELISA kit (R&D Systems, cat# DMC00B) per the kit instructions. Within each experiment, three separate wells were treated for each condition. Each data point represents a single well and graphical bars represent the mean of each group with error bars depicting the standard deviation. A one-way ANOVA with multiple comparisons was used to determine the statistical significance of differences between groups. Statistical analysis was performed with Prism 6 (GraphPad Software) and p values are 0.0003 (A1_low_ versus A1_med_) and 0.0001 for all other comparisons.

### Flow Cytometry for LPS Acute Neuroinflammation Model

Female C57BL/6 mice were dosed for 4 days with sCSF1R_inh_ at 8–10 weeks of age. One hour after dosing, mice were treated with LPS (1 mg/kg, i.p.; Sigma, cat# L6529). On day five, mice received sCSF1R_inh_ and were sacrificed 1 h later. Animals were perfused with ice cold PBS and microglia and infiltrating monocytes/macrophage populations were isolated as described above. The round-bottom 96-well plate containing cells was centrifuged at 2000 rpm for 1 min, and the supernatants were flicked out. Fifty microliters of mouse block were added to each well, mixed well with pipet tips and allowed to incubate for 10 min at room temperature. Twenty microliters per sample of the following antibody cocktail were added to the wells: CD11b-PCP Cy5.5 (1:100, BD Pharmingen catalog # 550993) and CD45-FITC (1:100, BD Pharmingen catalog # 553080).

Compensation control tubes were also set up with the beads and the individual antibodies. The antibodies and cells or beads were then incubated for 20 min at room temperature in the dark. Two hundred microliters of PBA were added to the cells in the staining plate, and the plate was centrifuged for 1 min at 2000 rpm. The cell pellets were resuspended with 230 µL MFF, mixed well with pipet tips and stored at 4 °C until run on the LSR. 20 µL counting beads were then added to each sample well for quantitation. The compensation beads were then washed by adding 1 mL PBA to each tube and were centrifuged at 2400 rpm for 5 min. The compensation control beads were resuspended with 250 µL PBA and stored until acquisition. Once sample acquisition was completed, sample results were analyzed using the FlowJo FACS analysis software (see Supplementary Fig. 5A for gating strategy).

### BrdU staining by flow cytometry

Spinal cords were homogenized in ice cold calcium and magnesium free HBSS using a glass dounce homogenizer. Homogenates were passed through a 70 µm cell strainer and centrifuged for 5 min at 2000 rpm, 4 °C. Pellets were resuspended in HBSS and washed once more in HBSS. Pellets were then resuspended in 30% percoll (GE Healthcare) at 4 °C and then centrifuged for 10 min at 700 *g* with the brake off. The supernatant was aspirated and the cells were resuspended and blocked in flow cytometry staining buffer containing mouse Fc block (Biolegend) on ice for 10 min. Cells were centrifuged for 5 min at 300 *g* at 4 °C. Cells were stained with primary antibodies against CX3CR1 (1:100, Biolegend catalog # 149022 Clone SA011F11), Ly-6C (1:100, Biolegend catalog #128037 clone HK1.4), CD45 (1:200, Biolegend catalog # 103106 Clone 30-F11), Ly-6G (1:100, Biolegend catalog #565964 clone 1A8), and CD11b (1:200, Biolegend catalog #101262 clone M1/70) for 30 min at 4 °C in the dark. Cells were washed once and then stained for BrdU using the Phase-flow kit (Clone 3D4, Biolegend catalog # 370706) according to the manufacturer’s instructions. Stained cells were acquired on a BD LSRII flow cytometer and analyzed using the FlowJo software (see Supplementary Fig. 5B–D for gating strategy).

### Cytokine/chemokine analysis in NOD-EAE spinal cord

Frozen spinal cord tissues were thawed, and 700 µl of Bioplex cell lysis buffer (BioRad catalog # 171-304011) containing factors 1 and 2 (protease and phosphatase inhibitors, respectively; BioRad catalog # 171-304012), and the protease inhibitor phenyl-methylsulfonyl fluoride (PMSF, 500 mM; Sigma-Aldrich) were added. Tissue was homogenized using zirconium beads and shaken on the Omni Bead Ruptor and then centrifuged at 4 °C at 6000 *g* for 20 min. The supernatant was removed and aliquoted. Cytokine/chemokine levels were then quantified via R&D Systems ELISA per the kit instructions (IP-10, cat#CY466; IL-6, cat# M6000B; IL-1β, cat# MLB00C; MCP1, cat# MJE00B; CSF1, cat#MMC00; IL12p40, cat# MP400; IL-10, cat# M1000B; RANTES, cat# MMR00; and TGF-β, cat#MB100B). The protein content of each sample was determined using the bicinchoninic acid (BCA) assay (Pierce cat # 23225, Rockford, IL), with bovine serum albumin (BSA) as a standard, according to the manufacturer’s protocol. Sample absorbance was read at 560 nm using a spectrophotometer (Molecular Devices Versa Max, Sunnyvale, CA). Results were normalized to protein concentration and cytokine/chemokine levels were reported as picograms of cytokine/mg of tissue protein. Each data point represents a single spinal cord sample and graphical bars represent the mean of each group with error bars depicting the standard error of the mean. A one-way ANOVA with multiple comparisons was used to determine the statistical significance of differences between groups. Statistical analysis was performed with Prism 6 (GraphPad Software) and p values are 0.0381 (IL-6), 0.0423 (IL-1β), and 0.0150 (MCP-1).

### Nanostring analysis

Nanostring analysis was performed as previously described^20,48^. Briefly, the MG550 NanoString chip was designed using the quantitative NanoString nCounter platform. Selection of genes was based on analyses that identified genes and proteins which were specifically or highly expressed in adult mouse microglia^48^ plus inflammation-related genes which were significantly affected in EAE, APP-PS1, and SOD1 mice. MG550 encompasses 400 unique and enriched microglial genes we have identified previously^48^ and additional 150 inflammation-, inflammasome- and phagocytosis-related genes. NanoString data were normalized and analyzed using the nSolver software. RNA ncounts were normalized using the geometric mean of six housekeeping genes (HKGs): *Cltc*, *Gapdh*, *Gusb*, *Hprt*, *Pgk1*, and *Tubb5*. A cutoff was introduced at the value of the highest negative control present on the chip. Fold changes were calculated using the average of each group. For each experiment, the fold changes were calculated comparing the experimental group to their appropriate controls. Each data point represents microglia from a single spinal cord sample and graphical bars represent the mean of each group with error bars depicting the standard error of the mean. A one-way ANOVA with multiple comparisons was used to determine the statistical significance of differences between groups. Statistical analysis was performed with Prism 6 (GraphPad Software) and *p* values are 0.0053 (TMEM119), 0.0203 (P2RY12), 0.0134 (CX3CR1), 0.0407 (SPP1), 0.0474 (Lilrb4), and 0.0011 (Fabp5).

### Silver staining

Spinal columns collected at Day 62 of the NOD-EAE were shipped to Mass Histology Services in Worcester, MA for decalcification, paraffin embedding and sectioning. A Bielschowsky’s Silver stain was performed as previously described to visualize nerve cells and fibers within the spinal cord^61^. Several sections from each individual mouse spinal cord were scored for the severity of axonal loss (*n* = 12 for vehicle, *n*-11 for 25 mg/kg, and *n* = 10 for 100 mg/kg). Graphical bars represent the mean pathology score of each group with error bars depicting the standard error of the mean. A Kruskal–Wallis nonparametric one-way ANOVA with multiple comparisons was used to determine the statistical significance of differences between groups. Statistical analysis was performed with Prism 6 (GraphPad Software) and the p-value is 0.0010.

## Supplementary information

Suppl Figure legends

Suppl Fig-1

Suppl Fig-2

Suppl Fig-3

Suppl Fig-4

Suppl Fig-5

Suppl Fig-6

Suppl Fig-7

Suppl Fig-8

Suppl Fig-9

Nanostring data set

NOD EAE sequencing file

Editable tables
