# Supplementary material for: CSF1R signaling is a regulator of pathogenesis in progressive MS
Source: Cell Death Dis. 2020 Oct 23;11(10):904. doi: 10.1038/s41419-020-03084-7 (PMC7584629; doi:10.1038/s41419-020-03084-7)
Supplement: Supplementary file 1 — Suppl Figure legends [file 41419_2020_3084_MOESM1_ESM.docx]

**Supplemental Figure Legends**

**Supplemental Figure 1: CSF1 transcript is elevated in murine models of neurodegenerative disease**

**(A)** Data table for human samples used in MS tissue analysis described in Figure 1 and Figure 5. All brain samples were taken from cortical white matter lesions. **(B)** *In situ* hybridization with CSF1R probe and immunohistochemistry with an IBA1 antibody reveals the presence of CSF1R transcripts within microglia. Scale bar: 50µm **(C)** IL-34 levels were not elevated in cerebral spinal fluid from Prog MS patients as compared with RRMS and CTL samples. Each data point represents an individual human sample while graphical columns represent the mean and standard error. Statistical significance was determined by a one-way ANOVA. **(D)** Multiple published *in vivo* and *in vitro* preclinical studies describe significant increases in CSF1 mRNA expression in different neurodegenerative and neuroinflammatory conditions such as Amyotrophic Lateral Sclerosis, Alzheimer’s Disease, and MS.

**Supplemental Figure 2:** **Additional characterization of the NOD-EAE spinal cord.**

**(A)** Flow cytometry gating for the quantification of microglia and infiltrating macropahges. Live, single cells were selected on the basis of forward and side scatter. Monocytes/macrophages (CD45^high^CD11b^+^) and resident microglia (CD45^low/int^CD11b^+^) were identified among CD45^+^ leukocytes in naïve (top) and EAE spinal cords (bottom). **(B)** RNA sequencing of spinal cord from different stages of the NOD-EAE model showing a heat map of gene expression changes in three different pathways: cytokines, toll like receptor (TLR), and triggering receptors expressed on myeloid cells/disease associated microglia (TREM2/DAM).

**Supplemental Figure 3: Pharmacokinetic properties of sCSF1R_inh_**

**(A)** Murine pharmacokinetic studies demonstrate that sCSF1R_inh_ shows good plasma and brain exposure and oral bioavailability in naïve animals across 24 hours. **(B,C)** Terminal plasma taken one hour after the last dose in both the LPS acute model and NOD-EAE model shows clear dose dependent sCSF1R_inh_ exposure.

**Supplemental Figure 4: sCSF1R_inh_ blocks IL-34 induced CSF1R signaling.**

**(A,B)** IL-34 (100 ng/mL) stimulation in BV2 microglia induces CSF1R and ERK1/2 phosphorylation after 5 minutes and sCSF1R_inh_ (500 nM) blocks these phosphorylation events **(C)** After 75 minutes of IL-34 stimulation, CSF1R is trafficked to the lysosome for degradation and sCSF1R_inh_ (500 nM) blocks CSF1R degradation. Data points represent quantification from a single Western Blot. Graphical columns represent the mean and standard deviation of biological triplicates. Statistical significance was determined by a one-way ANOVA and p values are indicated by **** p< 0.0001.

**Supplemental Figure 5: Characterization of PLX3397 in CSF1R inhibitor assays**

**(A)** CSF1 stimulation (100 ng/mL) induced MCP-1 release from primary murine microglia and PLX3397 blocked MCP-1 production **(B)** LPS (10 ng/mL) induced a significant increase in IL-12p40 production by primary murine microglia and PLX3397 significantly reduced IL-12p40. Data points represent the protein concentration per well and graphical columns represent the mean and standard deviation. Statistical significance was determined by a one-way ANOVA and p values are indicated by **** p< 0.0001. **(C)** Murine mixed glial cultures were treated with PLX3397 and Iba1 immunocytochemistry was used to identify microglia after 3 days. Quantitative analysis of Iba1^+^ area reveals that PLX3397 significantly depletes microglia in a concentration dependent manner. Data points represent the mean and standard deviation (n=3); the IC_50_ was calculated with Prism 6 (GraphPad Software). **(D)** Iba1 immunohistochemistry reveals a decrease in the number of cortical microglia in naïve C57BL/6 mice treated with vehicle versus PLX3397 for 7 days. Scale bar: 100 µm. Manual quantification of Iba1^+^ cells confirmed these qualitative observations. Data points represent the average Iba^+^ cell count per animal (n=2 images/animal, n=3-6 animals/group). Graphical columns represent the mean and standard error. Statistical significance was determined by a one-way ANOVA and p values are indicated by * p<0.05, ** p<0.01, and **** p< 0.0001. **(E)** Low magnification images of Iba1 immunohistochemistry in the cortex of C57BL/6 mice treated with either sCSF1R_inh_ or PLX3397 for 7 days. Scale bar: 100 µm. **(F)** Primary murine microglia were treated with PLX3397 and cell viability was assessed after 3 days using DAPI staining. PLX3397 treatment had no impact on microglial cell survival at the concentrations assessed in this experiment. Data points represent the number of DAPI^+^ cells per image (9 images/well) and graphical columns represent the mean and standard deviation of three wells.

**Supplemental Figure 6: Flow cytometry gating strategy for acute LPS model**

**(A)** For Sup Fig 6A-C, live cell gating was performed on the basis of forward and side scatter. Histograms of CD11b positivity were generated based upon the histogram of unstained cells. CD11b vs CD45 plots were then generated on the CD11b^+^ population from the histograms. **(B)** For Sup Fig 6D-F, live, single cells were selected on the basis of forward and side scatter. Leukocytes were gated on CD45 and neutrophils were excluded as CD11b^+^Ly6G^+^ cells. Next, CD11b^+^Ly6G^-^ cells were gated as either CD45^high^ monocytes/macrophages or CD45^low^CX3CR1^high^ microglia. **(C)** Staining controls for BrdU. **(D)** Histogram comparing CX3CR1 expression on mono/mac and microglia gates.

**Supplemental Figure 7: sCSF1R_inh_ reduces the number of microglia and macrophages *in vivo***

**(A)** Microglia and infiltrating macrophages isolated from the LPS acute model were analyzed by flow cytometry with gating based upon CD45 and CD11b immunoreactivity. The number of microglia as well as infiltrating macrophages is significant elevated in the model. **(B-C)** sCSF1R_inh_ significantly reduces the number of microglia and infiltrating macrophages in the spinal cord of the LPS acute model. Data points represent cell quantification from a pool of three animals. Graphical columns represent the mean and standard error of three different pools (n=9 animals/group). Statistical significance was determined by a one-way ANOVA and p values are indicated by **** p< 0.0001. **(D)** BrdU immunocytochemistry was used to identify proliferating microglia and infiltrating macrophages in the LPS acute model. Based upon CXCR3 and BrdU immunoreactivity, the number of proliferating microglia is significant elevated in the model. **(E-F)** sCSF1R_inh_ significantly reduces the number of proliferating microglia but did not impact macrophage proliferating in the spinal cord. Data points represent cell quantification from a single animal (n=6). Graphical columns represent the mean and standard error. Statistical significance was determined by a one-way ANOVA and p values are indicated by ** p<0.01, **** p< 0.0001.

**Supplemental Figure 8: sCSF1R_inh_ significantly suppresses clinical disease progression in the C57BL/6 EAE model**

**(A)** Disease scores demonstrate the typical course of paralytic symptoms in the C57BL/6 EAE model. Upon treatment with sCSF1R_inh_, mean disease scores were significantly reduced. Data points represent the mean and standard error (n=13). A two-way ANOVA was used to determine the statistical significance of the differences between vehicle and sCSF1R_inh_ treated group. For sCSF1R_inh_ at 25 mg/kg, p ≤ 0.0001 on Day 2 – Day 14 of treatment. **(B)** Area under the curve was calculated for the clinical disease course and sCSF1R_inh_ significantly reduces paralytic symptoms. Data points represent the area under the curve for each animal and graphical columns represent the mean and standard error. Statistical significance was determined by an unpaired T-test and p value is indicated by *** p< 0.001.

**Supplemental Figure 9: sCSF1R_inh_ significantly impacts microglial phenotypes in the NOD-EAE model.**

**(A)** Inflammatory cytokine production in the spinal cord was assessed with ELISA and sCSF1R_inh_ treatment at 25 mg/kg did not impact CSF1, IL12p40, RANTES, TNF-β, or IL-10. Data points represent the protein quantification from a single animal. Graphical columns represent the mean and standard error (n=3, 9, and 8 respectively). **(B)** Quantitative NanoString nCounter mRNA analysis of microglial gene expression in the spinal cord of the NOD-EAE model. Statistical significance was determined by a t-test and p values are indicated in the table.
