# Supplementary figures and images for: CSF1R signaling is a regulator of pathogenesis in progressive MS
Source: Cell Death Dis. 2020 Oct 23;11(10):904. doi: 10.1038/s41419-020-03084-7 (PMC7584629; doi:10.1038/s41419-020-03084-7)

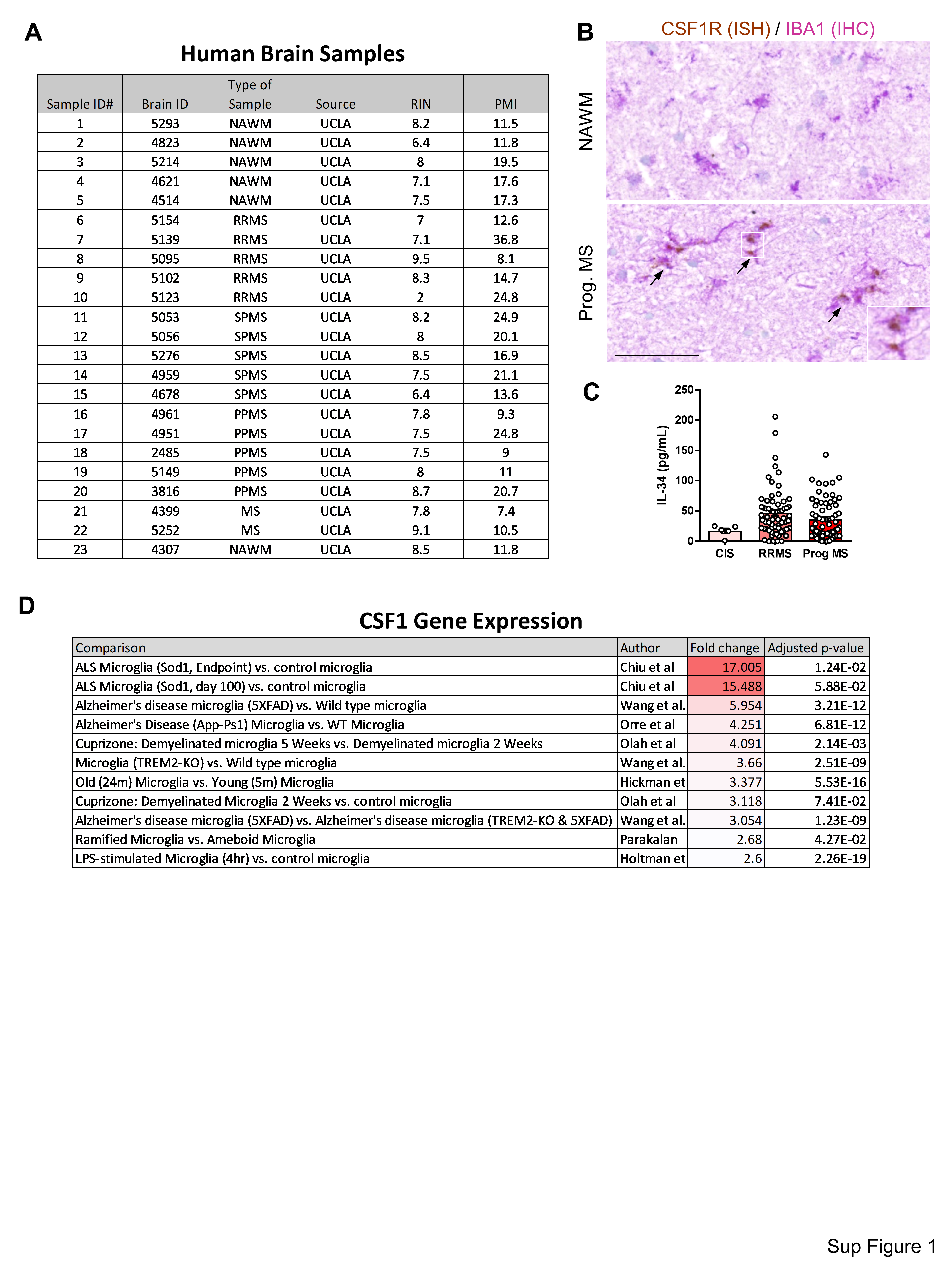

Supplement: Supplementary file 2 — Suppl Fig-1 [file 41419_2020_3084_MOESM2_ESM.tif]

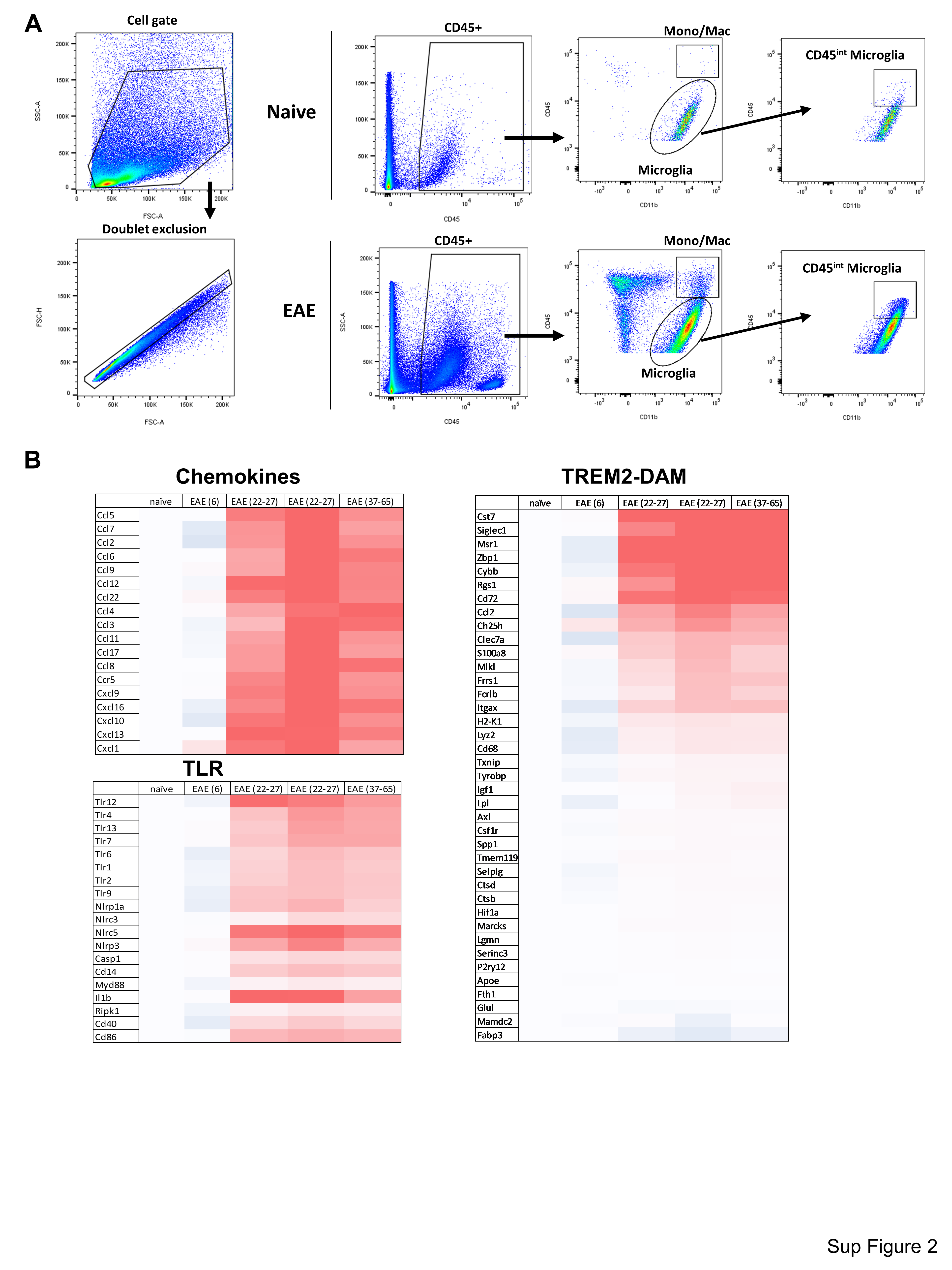

Supplement: Supplementary file 3 — Suppl Fig-2 [file 41419_2020_3084_MOESM3_ESM.tif]

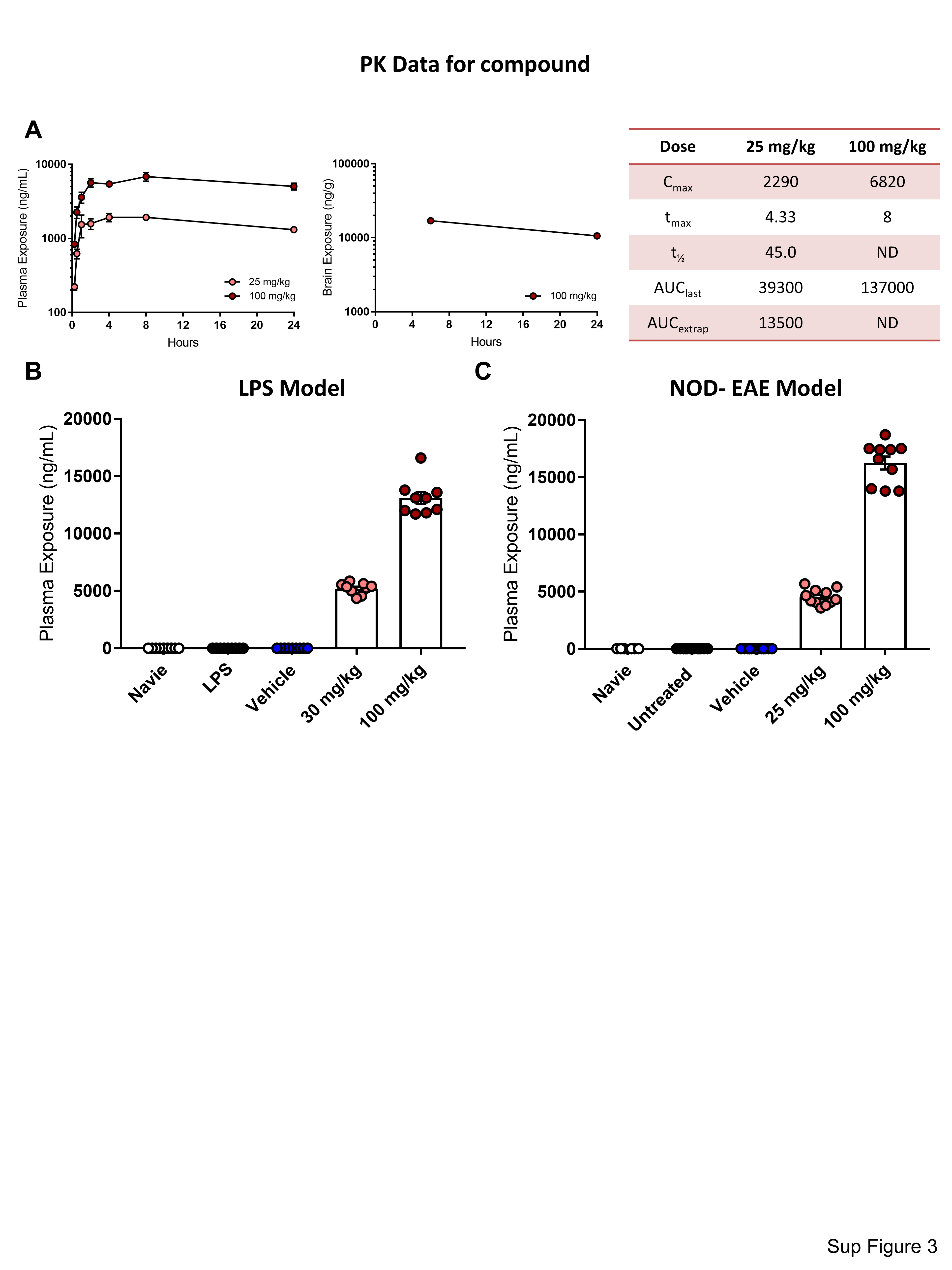

Supplement: Supplementary file 4 — Suppl Fig-3 [file 41419_2020_3084_MOESM4_ESM.tif]

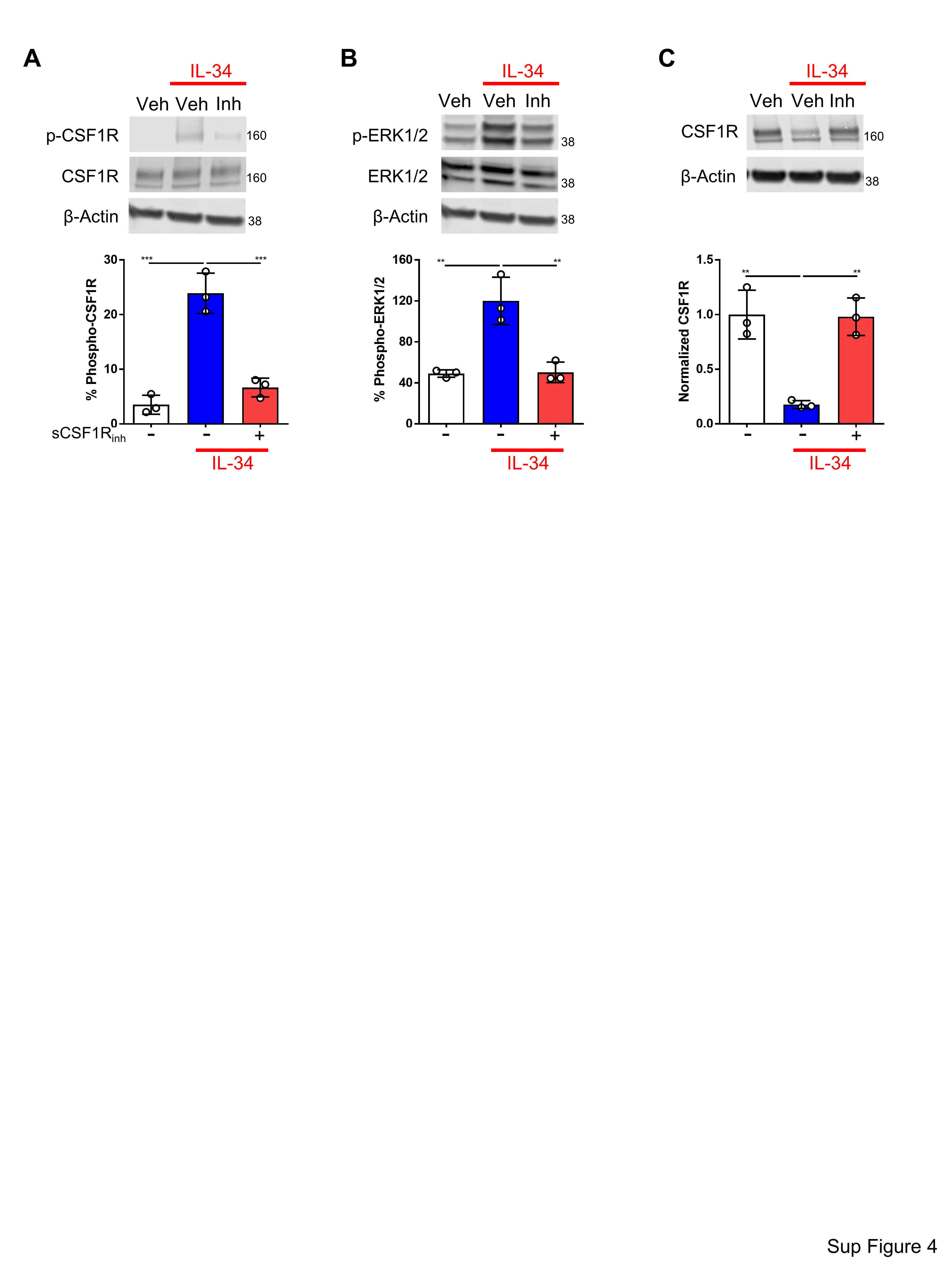

Supplement: Supplementary file 5 — Suppl Fig-4 [file 41419_2020_3084_MOESM5_ESM.tif]

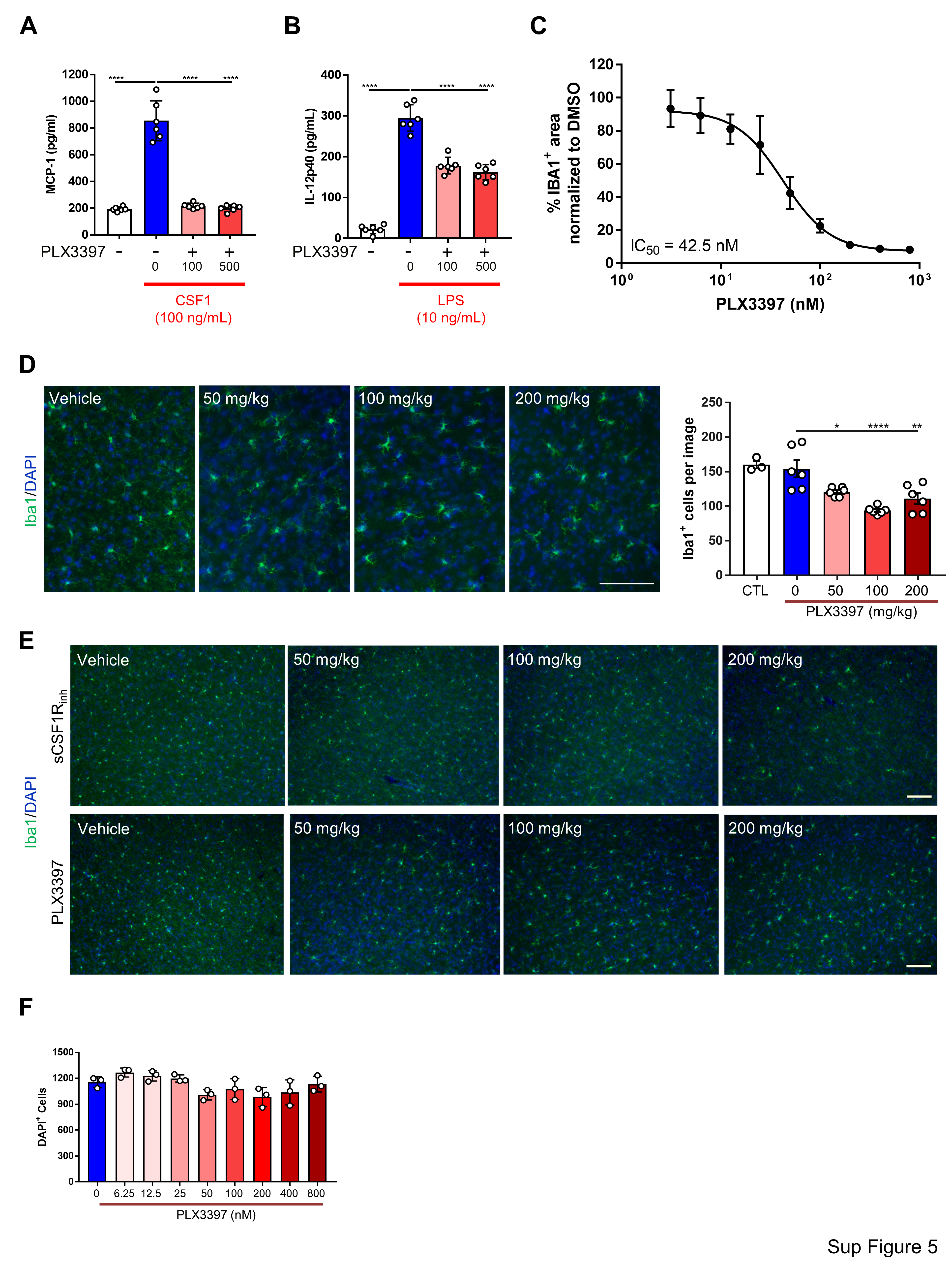

Supplement: Supplementary file 6 — Suppl Fig-5 [file 41419_2020_3084_MOESM6_ESM.tif]

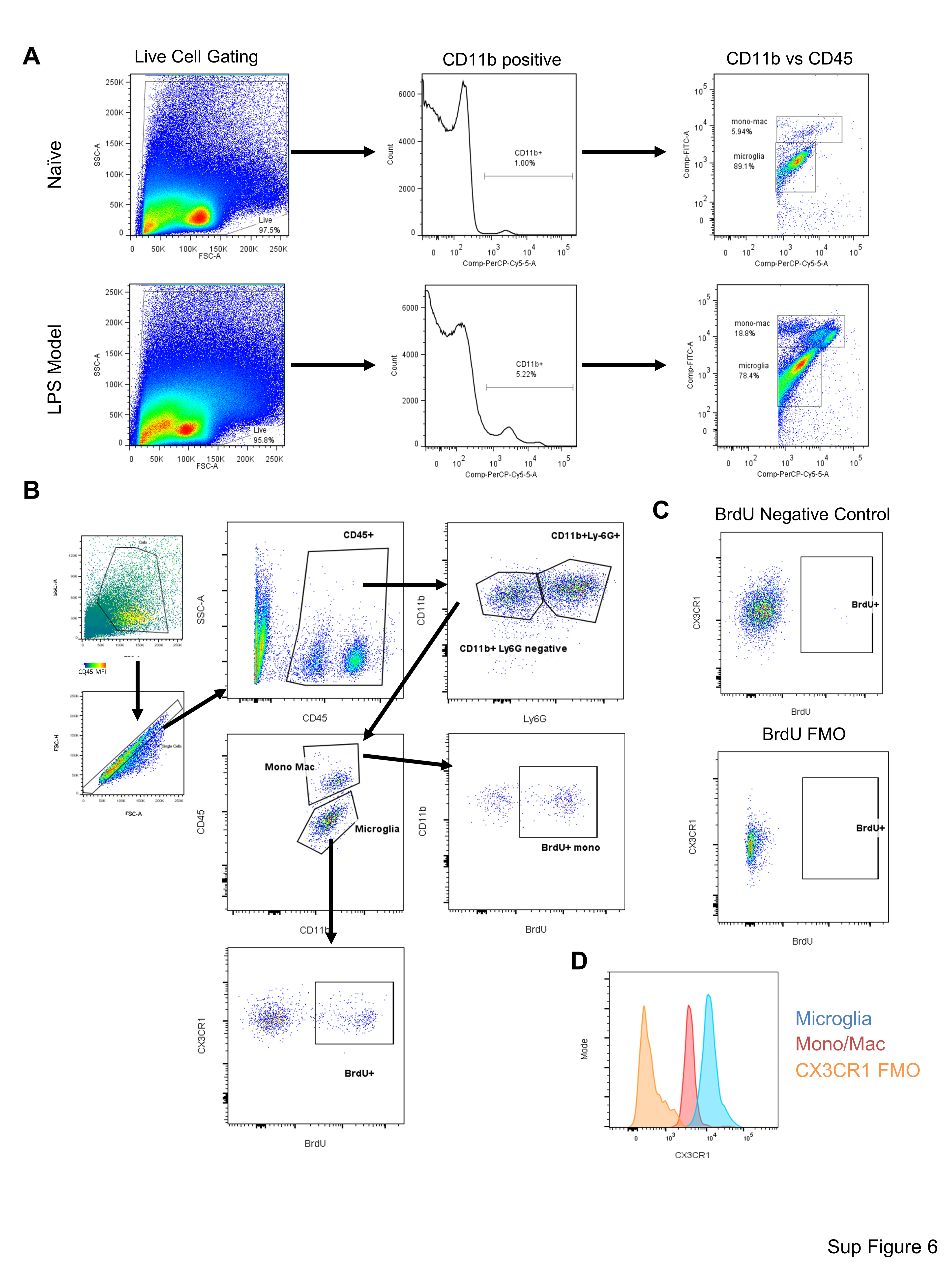

Supplement: Supplementary file 7 — Suppl Fig-6 [file 41419_2020_3084_MOESM7_ESM.tif]

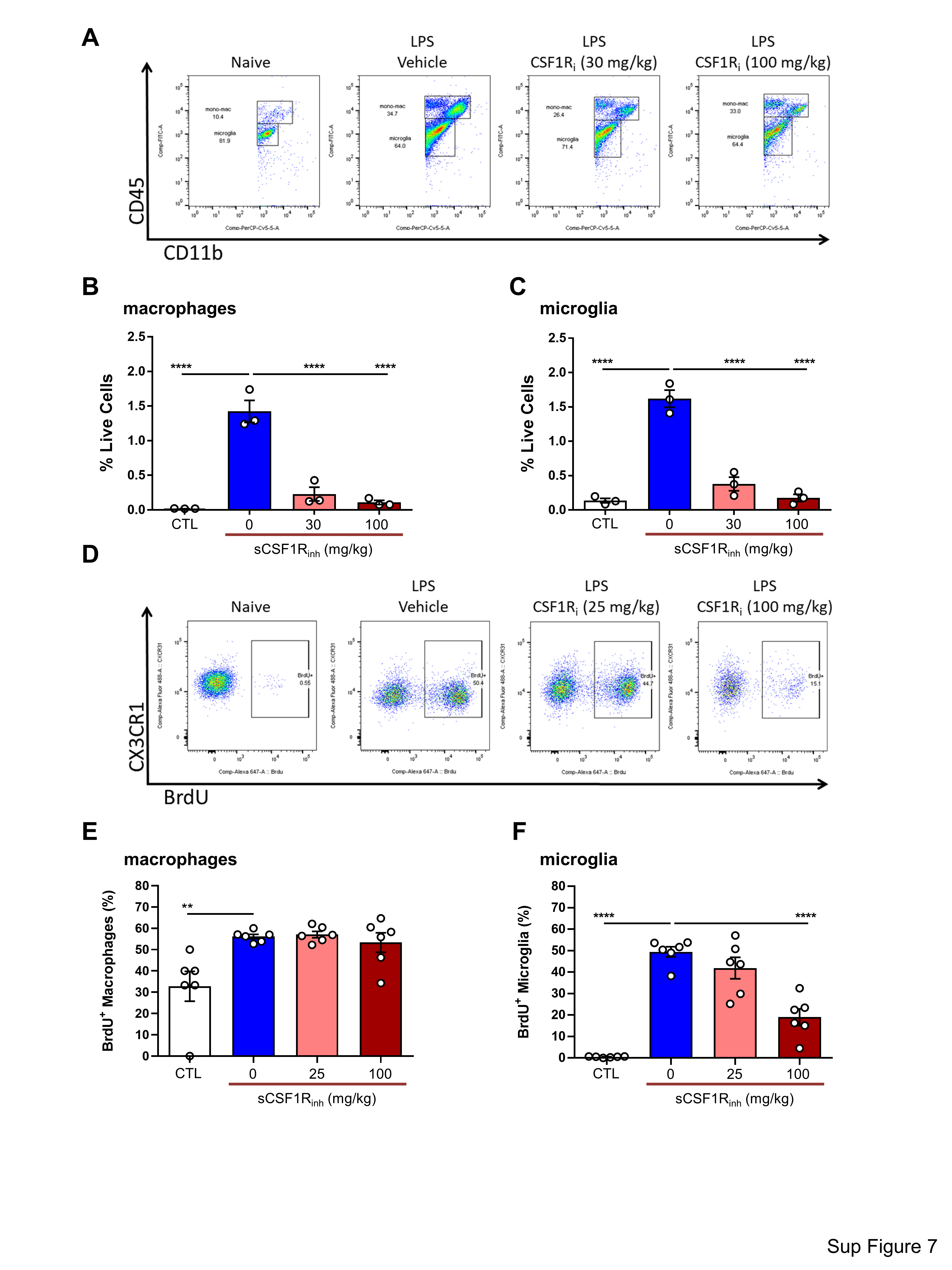

Supplement: Supplementary file 8 — Suppl Fig-7 [file 41419_2020_3084_MOESM8_ESM.tif]

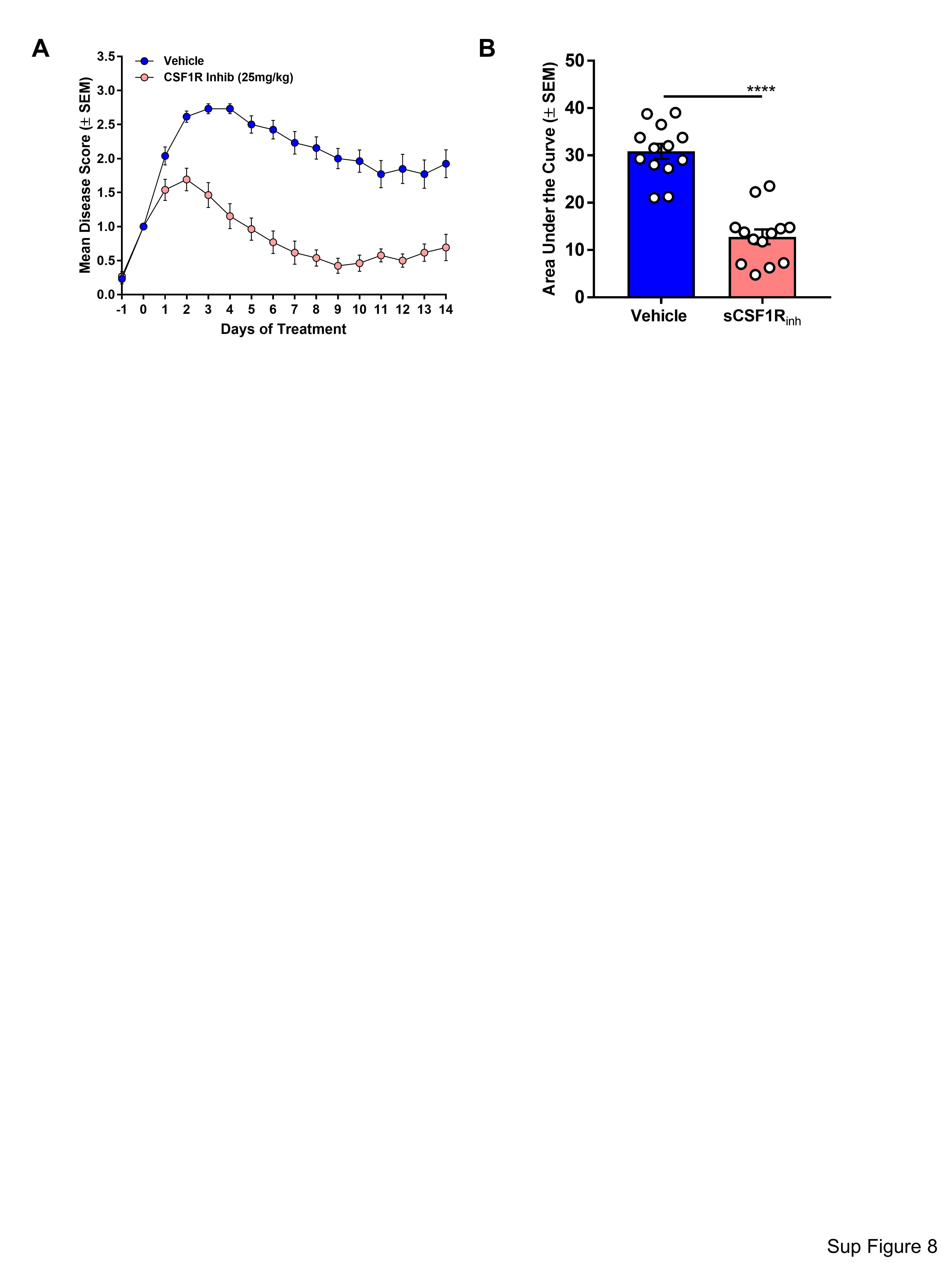

Supplement: Supplementary file 9 — Suppl Fig-8 [file 41419_2020_3084_MOESM9_ESM.tif]

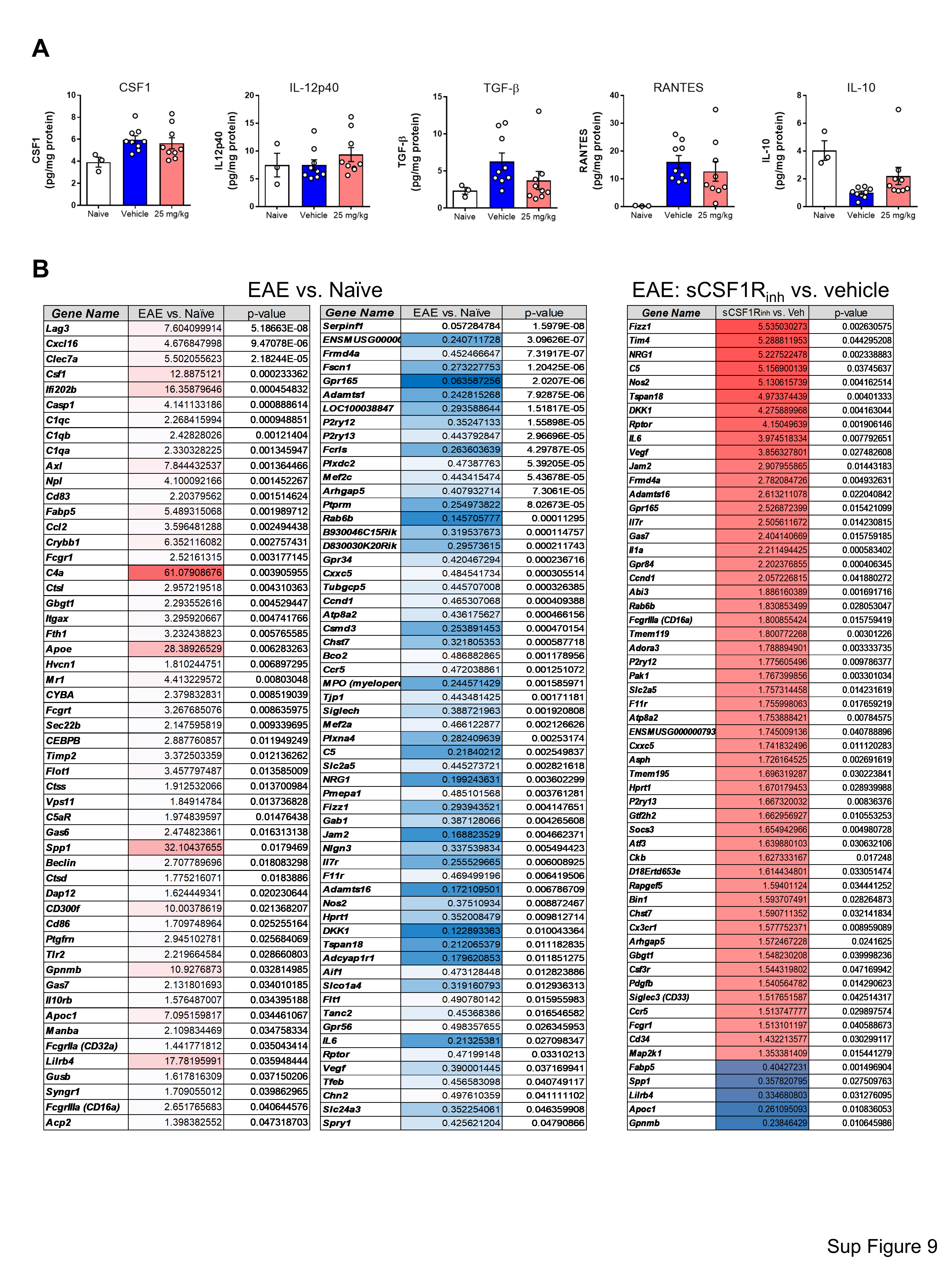

Supplement: Supplementary file 10 — Suppl Fig-9 [file 41419_2020_3084_MOESM10_ESM.tif]
